# Molecular Basis for Peptide Nitration by a Novel Cytochrome P450 Enzyme in RiPP Biosynthesis

**DOI:** 10.1021/acscatal.5c01932

**Published:** 2025-06-03

**Authors:** Katie Nolan, Remigio Usai, Bingnan Li, Stephanie Jordan, Yifan Wang

**Affiliations:** Department of Chemistry, 1355 University of Georgia, Athens, Georgia 30602, United States

**Keywords:** cytochrome P450 enzyme, aromatic nitration, substrate−protein interaction, crystal structure, spectroscopic characterization, rufomycin biosynthesis, ribosomally synthesized and post-translationally modified peptide (RiPP)

## Abstract

RufO is a unique cytochrome P450 enzyme (CYP) involved in the biosynthesis of rufomycin, an antituberculosis cyclic peptide featuring an unusual nitrated tyrosine. Recent studies have clarified RufO’s role in producing ribosomally synthesized and post-translationally modified peptides (RiPPs). Despite growing interest in nitrating enzymes and RiPP biosynthesis, the mechanism by which RufO recognizes and nitrates its pentapeptide substrate, MRYLH, remains poorly understood. In this study, we use a combination of spectroscopic, kinetic, and structural techniques to elucidate the molecular basis for peptide binding and heme-based nitration in RufO. Peptide binding is an endothermic process with a dissociation constant of 0.78 μM. Unlike most CYPs, RufO does not undergo the typical spin state conversion nor exhibit a significant increase in reduction potential upon substrate binding. The minimal perturbation to the heme center may lead to RufO’s lack of specificity for redox partners. However, significant shifts in the vibrational frequencies of carbonyl complexes upon substrate binding indicate a more polar heme distal site that favors a nonlinear binding conformation of diatomic gas molecules. These distinctive features contrast with TxtE, the only other CYP known to catalyze aromatic nitration. A 1.51 Å resolution crystal structure reveals that substrate binding induces significant conformational changes in the distal pocket, particularly in the regions interacting with Arg-2 and His-5 of the MRYLH peptide. While Tyr-3 is positioned similarly to its counterpart in P450_Blt_, a paralog that catalyzes peptide cross-linking, an extended hydrogen-bonding network constraining His-5 is unique to RufO and likely contributes to its distinct nitration activity. Furthermore, transient kinetic data suggest the sequential binding of O_2_ followed by ^•^NO and characterize a ferric-superoxo intermediate essential for the nitration activity. This study provides valuable insights into the substrate specificity and catalytic mechanisms of CYPs involved in nitration reactions and RiPP biosynthesis.

## Graphical abstract

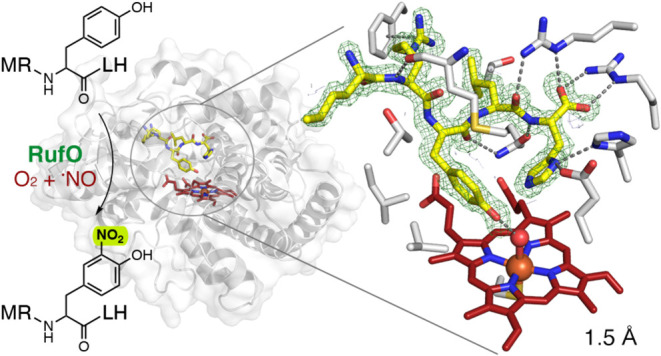

## Introduction

Ribosomally synthesized and post-translationally modified peptides (RiPPs) are a rapidly growing class of natural products with diverse bioactivities, including antimicrobial defense, signal transduction, and metal chelation.^1–3^ These peptides gain their structural and functional complexity through a variety of post-translational modifications (PTMs) catalyzed by specialized enzymes. Advances in genomic databases and bioinformatic tools have significantly accelerated the discovery of novel enzymatic activities in RiPP biosynthesis.^4^ Among the enzymes reported, cytochrome P450 enzymes (CYPs) have gained much attention.^5,6^ Traditionally known for their oxygenation functions,^7,8^ CYPs are increasingly recognized for their ability to catalyze a broad spectrum of reactions, particularly those involved in natural product biosynthesis. In RiPP biosynthetic pathways, CYPs facilitate not only oxygenation modifications, such as hydroxylation and epoxidation, but also promote non-oxygenation transformations such as C–C, C–O, and C–N bond formation to generate various macrocyclic scaffolds.^6,9–11^ The versatility and ubiquity of RiPP-synthesizing CYPs underscore their pivotal role in expanding the chemical diversity and bioactivities of the peptides. However, their functions can sometimes be misassigned, largely due to insufficient enzymatic characterization at the molecular level.

Recent studies on a nitrating CYP have showcased collective efforts to uncover a novel enzymatic function in RiPP biosynthesis. Nitration is a biologically significant transformation that modifies the function, stability, and reactivity of proteins and other biomolecules.^12–14^ While most biological nitration processes are known to proceed through less specific reactions involving peroxynitrite and other nitrative reactive species,^13,15,16^ biocatalytic nitration is rare and underexplored. The first example was reported for TxtE in thaxtomin biosynthesis, which catalyzes the direct nitration of l-tryptophan (Trp) in the presence of O_2_ and nitric oxide (^•^NO).^17^ This was followed by the identification of RufO in rufomycin biosynthesis and,^18,19^ more recently, Laj2 in lajollamycin biosynthesis that appears to be responsible for olefin nitration.^20^ Notably, these nitrating enzymes all belong to the CYP superfamily, which aligns with the engineering effort on the canonical CYP P450_BM3_, capable of nitrating aromatics and terminal aryl olefins when supplied with hydrogen peroxide and sodium nitrite.^21^ In the natural nitrating CYPs, ^•^NO is exclusively provided by an upstream nitric oxide synthase (NOS), such as RufN in the case of RufO. NOS-guided genome mining suggests the presence of additional biosynthetic gene clusters (BGCs) that may encode novel nitrated products.^20^ RufO was initially identified as an l-tyrosine (Tyr)-nitrating CYP, producing 3-nitro-l-tyrosine (3-NO_2_-Tyr) as a building block for nonribosomal peptide synthesis.^18,19^ However, spectroscopic and structural analyses indicated that RufO is unable to bind free Tyr to execute the anticipated nitration activity.^22,23^ Our crystal structure and molecular docking study revealed that RufO possesses a spacious heme distal pocket that could accommodate a peptide substrate, suggesting a new biological function for RufO.^22^ This catalytic enigma was later resolved when RufO was shown to nitrate a ribosomally synthesized pentapeptide.^24^ Figure 1 presents an up-to-date scheme of rufomycin biosynthesis, highlighting the unique nitration activity of RufO. Following the RufO-catalyzed nitration, the 3-NO_2_-Tyr moiety in the RiPP is cleaved out by an aminopeptidase (RufB) before entering the nonribosomal peptide synthetic pathway.^24^ Uncovering the catalytic role of RufO has broadened the catalytic repertoire of CYPs in RiPP biosynthesis. Furthermore, the discovery represents the first documented integration of a complete RiPP biosynthetic pathway into nonribosomal peptide synthesis.

**Figure 1:**
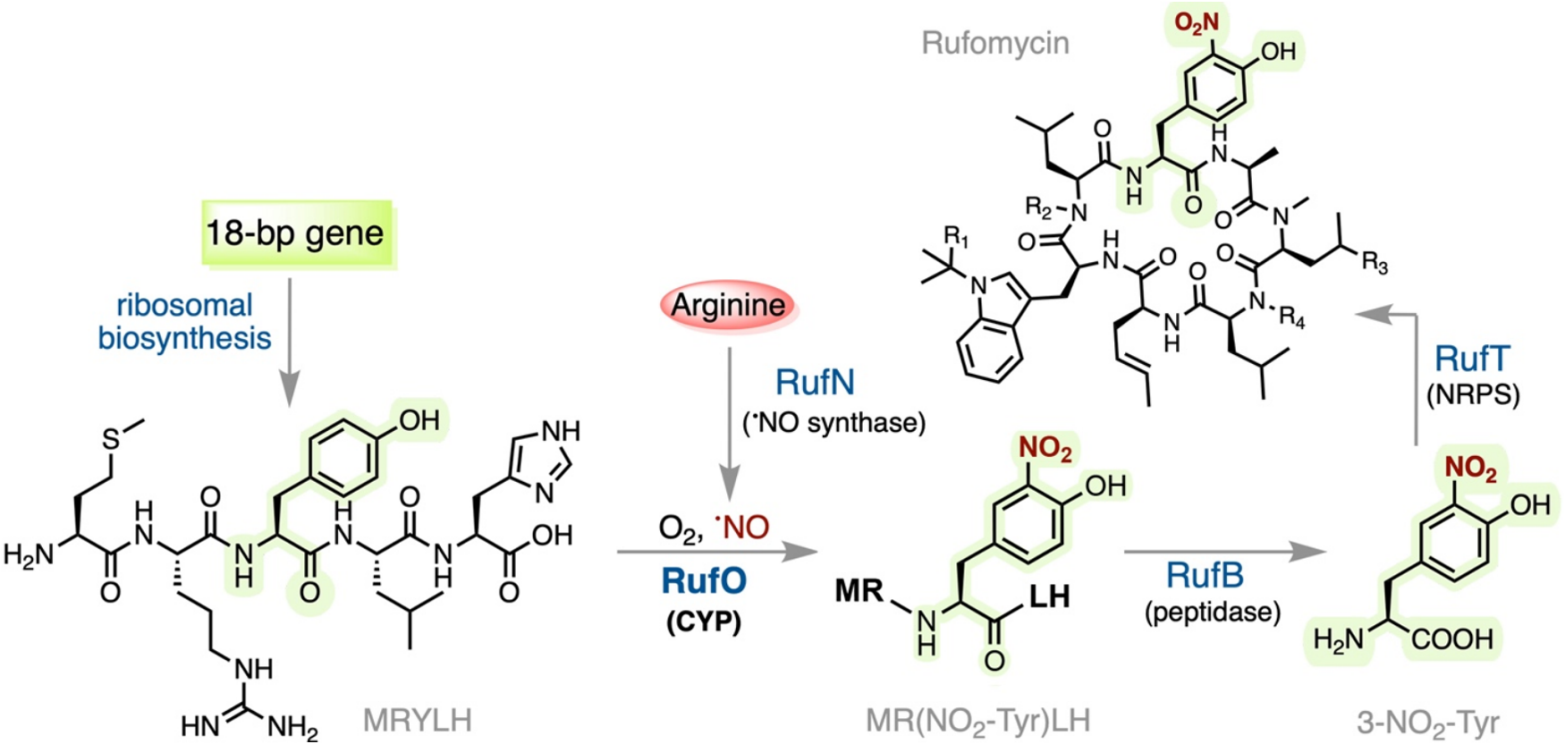
Rufomycin biosynthesis integrates the synthetic pathways of both ribosomal and nonribosomal peptides. RufO catalyzes tyrosyl nitration on a ribosomally synthesized pentapeptide encoded by an 18-bp gene. The nitric oxide synthase RufN supplies ^•^NO necessary for the reaction. Following the action of RufO, peptide cleavage is promoted by RufB to generate 3-NO_2_-Tyr as a building block for RufT, a nonribosomal peptide synthase (NRPS). R_1_-R_4_ indicate modifications present in various rufomycin analogs.

The newly identified pentapeptide substrate, MRYLH, is encoded by an 18-bp gene located upstream of the *rufNO* operon.^24^ This gene was not detected in the previously reported rufomycin BGC due to the challenges in characterizing such a small gene. Similar functional genes are found in hundreds of bacterial genomes, typically producing pentapeptides that feature a central Tyr and a *C*-terminal His.^25^ These genes are closely associated with CYP-encoding genes for RiPP production. Since the Tyr-His cross-linked RiPPs are among the first identified and most prevalent products, these CYPs were primarily thought to catalyze biaryl cyclization of the pentapeptides.^11^ The discovery of a nitrated RiPP from the rufomycin BGC once again illuminates the remarkable functions that CYPs have evolved to address specific metabolic needs. Interestingly, RufO shares 41.6% amino acid sequence identity with P450_Blt_, a paralogous CYP that catalyzes the formation of the Tyr–His cross-link in the same pentapeptide substrate. RufO can be transformed to catalyze the less efficient P450_Blt_ reaction, in addition to its native nitration activity, through just two active-site mutations in the I-helix.^24^ However, the reverse mutations in P450_Blt_, designed to mimic RufO, failed to support either cross-linking or nitration activity.^24^ These observations suggest that the molecular determinants underlying nitration versus cross-linking remain to be elucidated through detailed structural characterization and comparison.

Previous studies on RufO have employed spectroscopic and structural techniques to characterize its catalytic center, protein structure, and other biochemical properties.^22,23^ However, our current understanding is limited to its resting state, and there is a compelling need to characterize the enzyme in the context of the newly identified substrate. RufO adopts a protein fold typical of CYPs, but it exhibits significant differences in the surface regions that are critical for substrate recognition and catalytic selectivity.^22^ Little is known about RufO’s catalysis, including the substrate recognition mechanism, the changes at the catalytic site and overall protein structure in response to peptide binding, the interactions between the Cys-ligated heme center and small molecules, and the molecular details that dictate its peptide nitrating activity. While other RiPP-processing CYPs and the Trp-nitrating enzyme TxtE have been studied to varying extents, these enzymes primarily catalyze peptide cross-linking or free amino acid nitration, leaving a significant gap in our knowledge of CYP-dependent nitration chemistry in RiPP biosynthesis.

In this work, we apply various biophysical and biochemical techniques to investigate RufO in the presence of the peptide substrate, MRYLH, and an alternate substrate, Nle-RYLH, where norleucine (Nle) replaces methionine to eliminate the potential influence of sulfur oxidation. We characterize the changes in the heme center and protein conformation upon peptide binding, probe enzyme–substrate interactions, evaluate the effectiveness of commonly used CYP redox partners, analyze the transient kinetics of O_2_ and ^•^NO binding, and identify structural features that govern RufO’s nitration activity. The results are compared to those of other CYPs involved in aromatic nitration and peptide cyclization to provide a better understanding of the unique peptide-nitrating activity.

## Results

### Examining the Binding of Pentapeptide Substrates

Analysis of peptide binding to RufO was first performed via ultraviolet–visible (UV–vis) spectroscopy. RufO alone showed a pronounced Soret band at 421 nm and Q bands at 539 and 571 nm (Figure 2A). Upon titration of pentapeptide substrate, MRYLH, we observed a shift of the Soret band to 416 nm, accompanied by minor shifts of the Q-bands to 537 and 570 nm. The blueshift in the Soret band suggests Type-I binding, although the shift is less significant compared to typical Type-I binding events seen in CYPs,^26^ such as the shift to 390 nm in Trp-nitrating CYP TxtE (Figure S1A). Increasing the substrate-to-RufO concentration ratio above 1.3 did not further change the absorbance spectra (Figure S2), indicating that saturation was reached at relatively low substrate concentrations. To determine the dissociation constant (*K*_D_), the maximum peak-to-trough absorbance difference was plotted against the substrate-to-enzyme ratio (Figure 2B). The breakpoint indicated a binding stoichiometry of 0.89. However, due to the tight binding, neither the Hill nor the Morrison tight-binding models were able to provide a reasonable fit to derive a *K*_D_ value. The binding of an alternate substrate, Nle-RYLH (Figure S3A), was also tested to rule out any potential erroneous analysis resulting from methionine oxidation in the native substrate. Similar tight binding and spectral features were observed for Nle-RYLH (Figure S3B,C).

**Figure 2:**
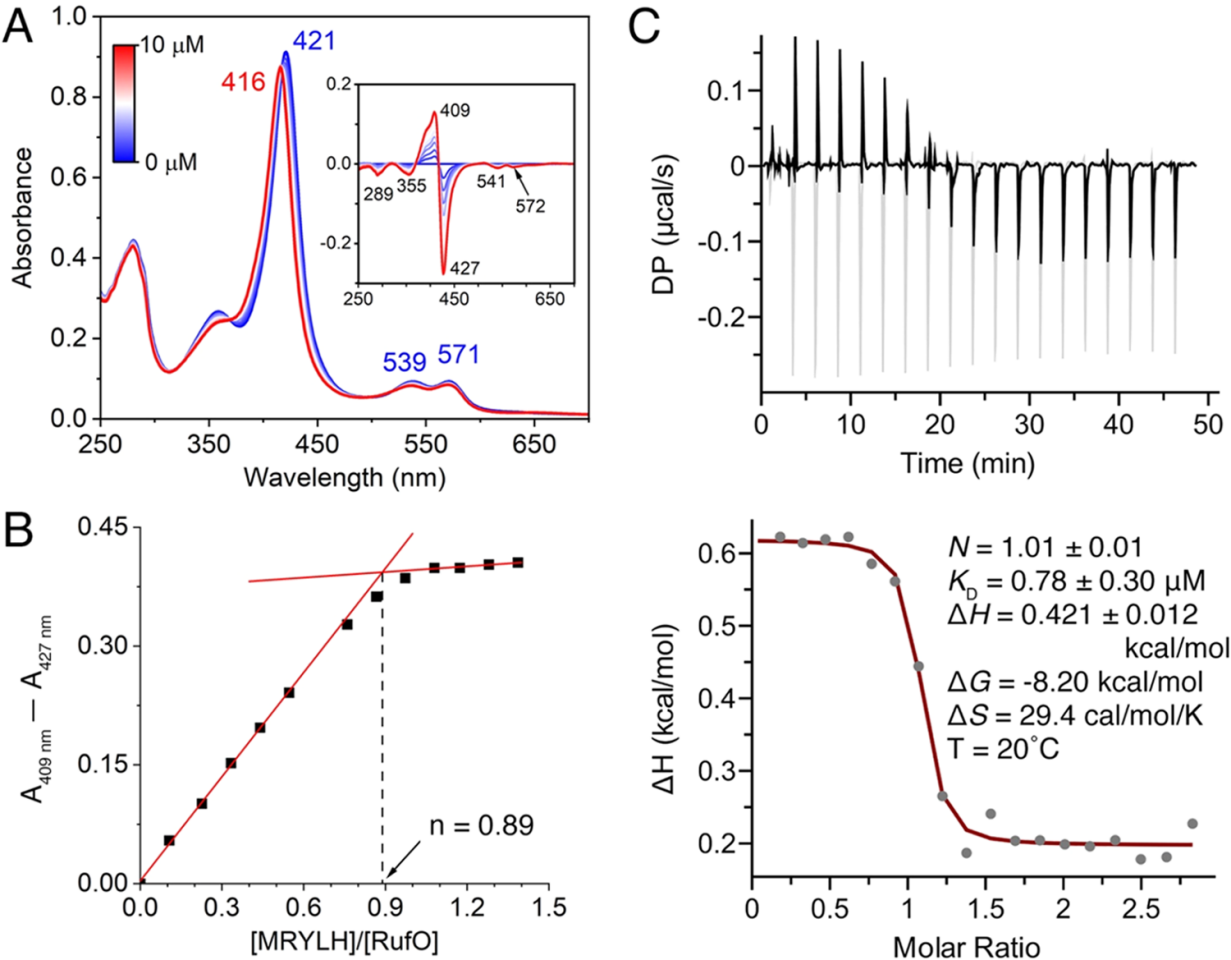
Titration analysis of peptide MRYLH binding to RufO. (A) UV–vis spectral changes upon titration of the peptide into 7.5 μM RufO, with the final concentration of the peptide reaching 10 μM. A transition from the resting-state (blue) to the peptide-bound RufO (red) was observed. The inset represents difference spectra. (B) Maximum difference in absorbance (*A*_409_–*A*_427_) plotted against the concentration ratio of peptide to RufO. The linear fitting (red lines) indicates a binding stoichiometry of 0.89. (C) The upper panel shows the changes in differential power observed with successive injections of 2.8 mM peptide to 190 μM RufO (black) and the control with 2.8 mM peptide into buffer (gray). Both traces have been baseline-corrected. The lower panel displays the integrated enthalpic changes for each injection after control subtraction, resulting in a fit consistent with a single binding site (red line).

Isothermal titration calorimetry (ITC) was used to more accurately determine the *K*_D_ and further assess the thermodynamics of the peptide binding process. The calorimetric titrations revealed an overall endothermic binding event (enthalpy change Δ*H* = 0.421 kcal/mol), after accounting for the control titration of the peptide to the buffer (Figure 2C). This observation suggests an entropy-driven binding event, likely resulting from the displacement of solvent molecules at the binding interface or significant conformational changes in the protein. This result aligns with the crystal structure of resting-state RufO, which reveals many water molecules present in the distal pocket and large unresolved surface regions.^22^ The resulting Δ*S* of 29.4 cal/mol/K can be attributed to the disruption of water clathrate interactions surrounding the nonpolar distal site of RufO, which outweighs the loss of conformational entropy associated with the ordering of the dynamic regions upon peptide binding. The integrated enthalpic changes were well fit to a single binding site model, yielding a *K*_D_ of 0.78 μM. The endothermic binding was also observed for Nle-RYLH (Figure S3D), which showed a comparable binding affinity (*K*_D_ = 1.60 μM) but a larger Δ*H*. The less rigid and smaller side chain of Nle, as compared to Met, may contribute to the difference in Δ*H* when forming the complexes. The tight binding of the peptide substrates in RufO contrasts with Trp binding in TxtE, which exhibits an exothermic binding event (Δ*H* = −14.6 kcal/mol) with a weaker binding affinity (*K*_D_ = 16.0 μM) (Figure S1B). Substrate binding in TxtE induces less conformational changes than in RufO due to the smaller size of Trp, and the enzyme–substrate interaction is primarily driven by hydrogen bonding and polar contacts, as revealed by its crystal structures.^27^ With a negative Δ*S*, it appears that TxtE does not undergo significant desolvation when forming the substrate-bound complex, in contrast to RufO. The observed differences in binding affinities between RufO and TxtE can be attributed to the disparity in cellular concentrations of their respective substrates. The pentapeptide, which is ribosomally regulated, is present at a lower concentration, whereas Trp is a ubiquitous metabolite.

### Probing the Changes of the Catalytic Center upon Peptide Binding

Given that the substrate binding in RufO is distinct from that of TxtE and other CYPs, we used electron paramagnetic resonance (EPR) and resonance Raman (rR) spectroscopies to investigate how substrate influences the heme center in these enzymes. The X-band continuous-wave EPR spectrum of RufO revealed a rhombic low-spin (*S* = 1/2) species with *g*-values at 2.42, 2.25, and 1.93 (Figure 3A), which is characteristic of resting state CYPs due to the coordination of a distal water ligand.^28^ In typical CYPs, substrate binding displaces the water ligand, converting the heme from low to high spin (*S* = 5/2). However, when 2 mM MRYLH or Nle-RYLH was added to RufO, the EPR spectrum exhibited a more rhombic, low-spin species with *g*-values at 2.54 (2.55 for Nle-RYLH), 2.24, and 1.88. Quantitative analysis suggests that 84% of the original low-spin species was converted to the new low-spin species. Even though tight substrate binding is expected, freezing artifacts during EPR sample preparation could contribute to the residual substrate-free signals. A minor species with *g*_max_ at 2.48 in the RufO alone sample (marked by an asterisk) has been consistently observed,^22^ which may arise from minor conformational heterogeneity of the heme center or a freezing artifact. It presumably transitioned to the species with *g*_max_ at 2.58 in the peptide-bound spectra. Additionally, no high-spin signal was observed, suggesting that the distal water ligand was not displaced. Instead, subtle changes in the heme environment were detected, indicating that while the peptides bind at the distal site, they do not significantly perturb the electronic structure of the heme center.

**Figure 3:**
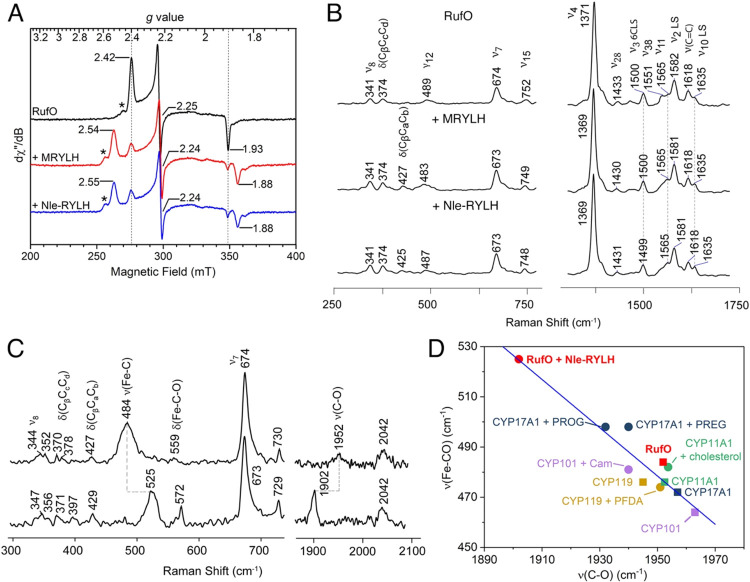
Spectroscopic characterization of the RufO heme center with and without peptide substrates. (A) X-band continuous wave EPR spectra of RufO (black), RufO with 2 mM MRYLH (red), and RufO with 2 mM Nle-RYLH (blue), measured at 10 K and 1 mW microwave power. The minor species marked by asterisks likely arise from conformational heterogeneity of the heme center or a freezing artifact. (B) rR spectra of RufO (top), RufO with 2 mM MRYLH (middle), and RufO with 2 mM Nle-RYLH (bottom). The spectra were collected using a 405 nm excitation laser at 5 mW. (C) rR spectra of CO adducts of ferrous RufO (top) and ferrous RufO with 2 mM Nle-RYLH (bottom). Spectra were collected using a 442 nm excitation line at 1 mW. (D) Backbonding correlations between *v*(Fe–C) and *v*(C–O) stretching frequencies for various CYPs in the absence (squares) and presence (circles) of ligands. Data points for RufO from this study are highlighted in red.

The binding of MRYLH and Nle-RYLH was also probed by rR spectroscopy. In both the high- and low-frequency regions, minor spectral differences were observed between RufO alone and RufO-peptide complexes (Figure 3B). The oxidation marker band ν_4_ appeared at 1371 cm^–1^ and shifted down to 1369 cm^–1^ upon peptide binding. The core size marker bands, ν_3_, ν_2_, and ν_10_, which are sensitive to spin state and coordination, showed minimal shifts, indicating a six-coordinate low-spin (6CLS) heme that is not sensitive to peptide binding. This observation is consistent with our EPR results. Additional notable changes include the disappearance of the ν_38_ band at 1551 cm^–1^ and the occurrence of δ­(C_β_C_a_C_b_) at 427 cm^–1^, suggesting subtle alterations in the heme structure caused by peptide introduction. In contrast, rR spectra of TxtE displayed significant changes upon Trp binding (Figure S1C). The ν_4_ band, initially appeared at 1374 cm^–1^, shifted down to 1373 cm^–1^ and dramatically decreased in intensity upon Trp binding. The ν_3_ band, initially at 1504 cm^–1^ and indicative of a 6CLS complex, downshifted to 1488 cm^–1^, suggesting a conversion to a five-coordinate high-spin (5CHS) heme. This spin-state conversion was also confirmed by a shift in ν_2_ from 1588 to 1570 cm^–1^ and the disappearance of the low-spin ν_10_ at 1640 cm^–1^. These spectral changes are typical in many CYPs,^29,30^ making the minimal shifts observed in RufO unusual. The contrasting responses of RufO and TxtE to substrate binding indicate that spin-state conversion is not a requirement for heme reduction and the subsequent nitration activity. Spectroscopic characterization of CYPs involved in RiPP biosynthesis is limited, and our observations offer valuable insights for analogous systems. Similar to RufO, other CYPs targeting peptide substrates may exhibit minimal changes to the heme center when interacting with their bulky substrates, potentially leading to alterations in some early steps of the standard CYP catalytic cycle.

To further characterize the interactions between the peptides and heme-bound small molecules, we generated carbonyl complexes for rR measurements (Figure 3C). Shifts in frequencies and intensities associated with the Fe–C–O fragment reveal important details about steric and electronic changes in the distal pocket. Key vibrational modes include the ν­(Fe–C) stretching modes (typically in the range of 460–490 cm^–1^ in CYPs), the ν­(C–O) stretching modes (1920–1970 cm^–1^), and δ­(Fe–C–O) bending modes (550–570 cm^–1^).^31^ The substrate-free form of RufO showed its ν­(Fe–C) stretching at 484 cm^–1^, toward the high end of the typical CYP range. Upon peptide binding, the ν­(Fe–C) mode exhibited a dramatic upshift to 525 cm^–1^, accompanied by a downshift in ν­(C–O) from 1952 to 1902 cm^–1^. The inverse correlation between ν­(C–O) and ν­(Fe–C) suggests a strengthened Fe–C bond and a weakened C–O bond. These shifts align well with a backbonding correlation trend line established by other CYPs,^32^ though the peptide-bound RufO is located at the extreme end (Figure 3D). The pronounced shifts of 41 cm^–1^ in ν­(Fe–C) and 50 cm^–1^ in ν­(C–O) modes are likely due to a more polar environment introduced by the peptide.^33^ Furthermore, peptide binding appears to induce steric hindrance, resulting in a bent CO conformation, as evidenced by the increased resonance enhancement and significant upshift of the δ­(Fe–C–O) mode from 559 to 572 cm^–1^.^34^ This shift typically only occurs by a few cm^–1^ in other CYPs.^31–33,35^ The result suggests that peptide binding may reshape the active site of RufO to favor binding nonlinear gas molecules. While CO bending generally reduces π-backbonding from the iron, it can lower the energy of the CO π* orbital, enhancing its overlap with both the π and π* orbitals of the porphyrin.^34^ This increased overlap strengthens vibrational coupling between the CO and the porphyrin, leading to an increase in the ν­(Fe–C) stretching frequency. Additionally, bending of the Fe–C–O unit would facilitate mixing between the ν­(Fe–C) and δ­(Fe–C–O) modes, leading to an upshift in the ν­(Fe–C) and a corresponding downshift in the ν­(C–O).^36^

### Evaluating the Effectiveness of Various Redox Partners in Nitration Reactions

The distinct characteristics of RufO among CYPs prompted us to investigate its catalytic efficiency with various redox systems. Nitrating CYPs require only a single electron transfer from an external redox partner, in contrast to the canonical CYP mechanism, which involves two electron transfers.^37,38^ It is proposed that different redox partners and oxidants can influence the product distribution in CYP catalysis, and, in some cases, new products have been observed by screening with various redox pairs.^39–41^ Additionally, interactions between TxtE and a reductase have been shown to modulate the enzyme’s activity and regioselectivity.^42,43^ Thus, we selected three redox partners commonly used in CYP catalysis to reconstitute nitration activity in RufO, comparing its efficiency with that of TxtE. Two of the selected systems, putidaredoxin (PdX) and spinach ferredoxin (SpFdX), are [2Fe-2S] cluster-containing ferredoxins that are paired with their respective reductase proteins, PdR and SpFdR. These redox partners are similar to PuX/PuR, the bacterial redox pair previously used in RufO activity assays.^24^ The third system, cytochrome P450 reductase (CPR), contains an FAD-binding domain and transfers electrons via an FMN-containing domain to subsequently reduce CYPs.

In the presence of excess ^•^NO, RufO catalyzes nitration of the peptide substrate under aerobic conditions, resulting in measurable product formation with each of the tested redox pairs (Figure 4A). The nitrated MRYLH product exhibited absorbance peaks at 278 and 359 nm, appeared bright yellow in color, and showed multiple protonated ions in high-resolution mass spectrometry analysis (Figure 4B and 4C). Experimental and theoretical *m*/*z* values for these ions and their isotope patterns are shown in Figure S4. Additionally, activity assays with Nle-RYLH and subsequent product analysis also confirmed the nitration activity of RufO (Figure S5). Control experiments demonstrated that RufO-catalyzed nitration depends on both O_2_ and ^•^NO, and free heme alone could not catalyze peptide nitration (Figure S6). The highest conversion rate was observed in the reaction using Nle-RYLH and SpFdX/SpFdR, which was set as 100% for normalization. Although the experimental conditions were not optimized for the maximum activities, nitrating enzymes are generally known for their low *in vitro* efficiency, likely due to the low coupling efficiency and the potential absence of their associated ^•^NO synthases.^42^

**Figure 4:**
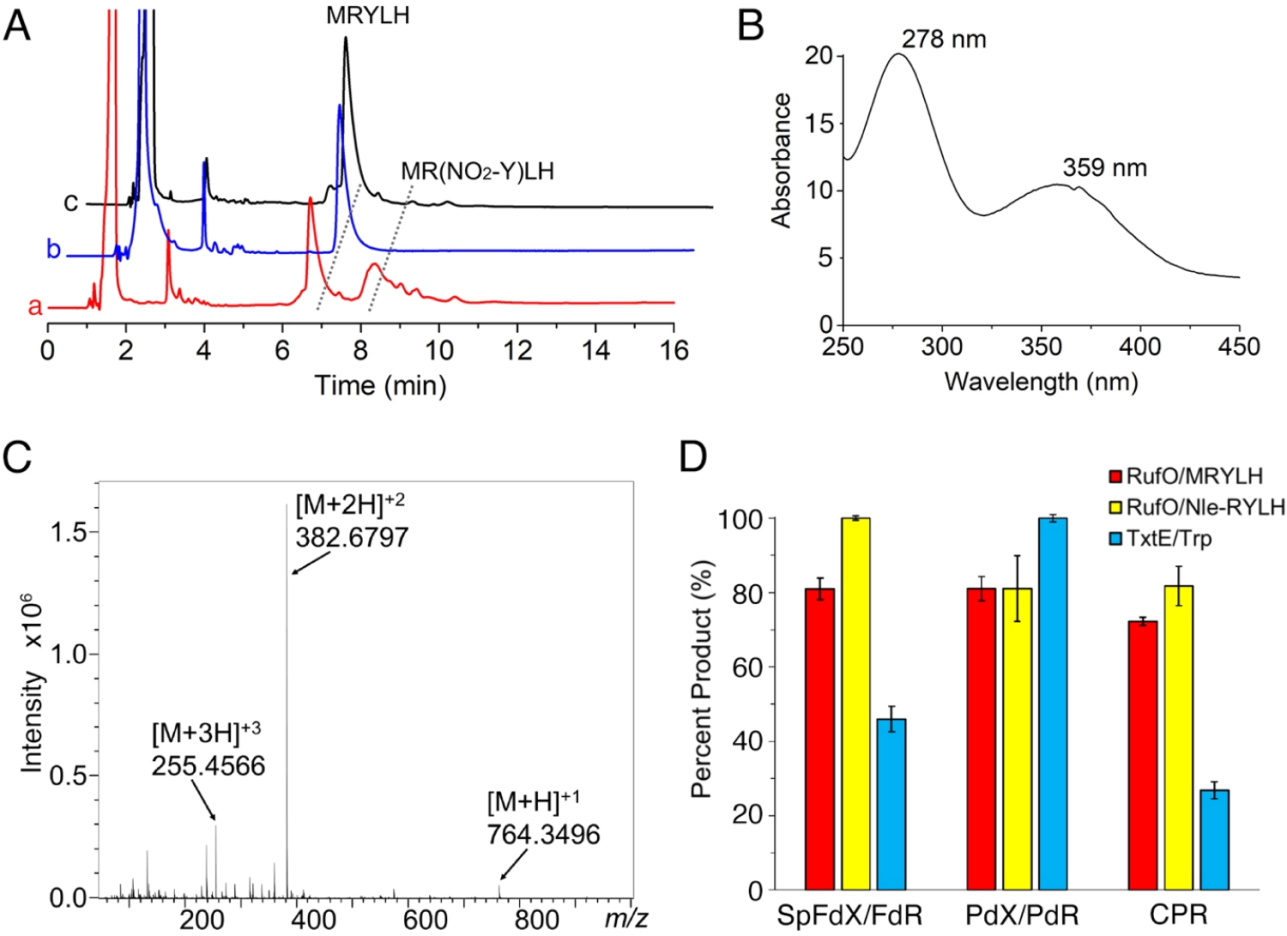
Activity assessment of nitrating CYPs. (A) HPLC profiles of (a) a reaction containing 20 μM RufO, 1 mM MRYLH peptide, 1 mM NADH, 40 μM CPR, and 1 mM DEA NONOate in the presence of O_2_, (b) a control reaction without ^•^NO addition, and (c) a control reaction without RufO addition. The pentapeptide and nitrated product, MR­(NO_2_–Y)­LH, eluted at 6.5 and 8.0 min, respectively. All traces were monitored at an absorbance wavelength of 280 nm. (B) Absorbance spectrum and (C) high-resolution mass spectrum of the product. MR­(NO_2_–Y)­LH exhibits absorbance peaks at 278 and 359 nm. In the positive ion mode, three pronated ions derived from the product were observed. (D) Product formation by RufO and TxtE reactions with various redox systems. RufO reactions were carried out with both MRYLH and Nle-RYLH peptides, while TxtE was reacted with Trp. The reaction with the highest product yield for each enzyme was normalized to 100% for comparison across different redox systems.

Bacterial CYP catalysis often relies on a redox potential gradient involving multiple components including ferredoxins and their reductases.^40^ CYPs maintain relatively negative potentials in the resting state to avoid uncoupled reactions. An increase in reduction potential upon substrate binding facilitates reduction of the heme and triggers catalysis. A feature of this carefully constructed gradient is a preference for a particular redox partner. TxtE exhibited this typical CYP behavior, showing a clear preference for PdX/PdR (Figure 4D). In the case of RufO, comparable conversion rates were observed across all assays, indicating a lack of specificity for electron transfer proteins. This could be attributed to the retention of a low-spin heme upon substrate binding, as evidenced by the EPR and rR data. To further explain this unusual observation, the reduction potential of RufO was measured in the presence of the substrate. The reduction potential of resting state RufO was previously determined and falls within the typical range for CYPs with a value of −325 mV.^22^ Using a dye-coupled assay,^44^ we observed that substrate binding increased the reduction potentials to −297 and −289 mV for MRYLH and Nle-RYLH, respectively (Figure S7). Typical CYPs have their reduction potential increased by approximately 100 mV upon substrate binding.^45^ This small change in reduction potential aligns with the spectroscopic evidence indicating minimal perturbations to the heme center upon substrate binding and further underscores RufO’s distinct characteristics compared to other CYPs. Despite the small increase in redox potential, RufO still underwent slow heme reduction upon incubation with redox proteins under anaerobic conditions, as evidenced by its absorption spectral changes (Figure S8). The lack of strong preference for the tested reductases indicates that RufO’s catalysis may be regulated by factors beyond redox potential shifts.

### Transient Kinetic Study of O_2_ and ^•^NO Binding to the Heme Center

TxtE was reported to bind O_2_ prior to *^•^* NO in the productive pathway of Trp nitration.^37,38^ To determine whether RufO follows a similar small-molecule activation pathway and forms analogous intermediates, we investigated transient kinetics of O_2_ and ^•^NO binding to ferrous RufO using stopped-flow spectroscopy in both ligand-free and peptide-bound states.

The rapid mixing of peptide-bound ferrous RufO with O_2_ resulted in the formation of a new species with absorbance peaks at 358, 432 (Soret), 558, and 585 nm (Figure 5A). These spectral shifts are consistent with ferric-superoxo (Fe^III^–O–O^•^ *^–^*) intermediates reported in TxtE and other CYPs, where the Q bands generally feature a maximum around 550–560 nm and, in some cases, a shoulder around 580 nm.^37,38,46^ This superoxo intermediate peaked at approximately 0.05 s and remained stable for up to 100 s before gradually decaying. A similar intermediate was also observed when ferrous RufO was mixed with O_2_ in the absence of the peptide, though with a smaller shift of the Soret peak to 428 nm (Figure S9A). To determine the second-order rate constants for O_2_ binding, rapid-mixing experiments were conducted using buffers with varying O_2_ concentrations. The formation rate of the intermediate exhibited a linear correlation with O_2_ concentration, yielding an association rate constant (*k*_1_) of 3.55 μM^–1^ s^–1^ for RufO alone and 3.83 μM^–1^ s^–1^ for the substrate-bound complex (Figure 5B). These values are comparable to the reported *k*_1_ of 4.43 μM^–1^ s^–1^ for Trp-bound TxtE and are considered rapid, given that O_2_ binding in most CYPs typically falls within the range of 0.1–10 μM^–1^ s^–1^.^47^ While the association of O_2_ appears unaffected by peptide binding, the y-intercept from the linear fitting, which approximates the dissociation rate constant (*k*_2_), decreases from 54.4 to 6.5 s^–1^ upon peptide binding. This suggests that the presence of substrate stabilizes the ferric-superoxo intermediate, which possibly occurs via the hydroxyl group of Tyr-3 that rests near the heme ligand (see the crystal structure below). A similar stabilizing effect of substrate binding on the ferric-superoxo intermediate may also be credible in TxtE, given the close proximity of its substrate indole amine to the iron center.^27^

**Figure 5:**
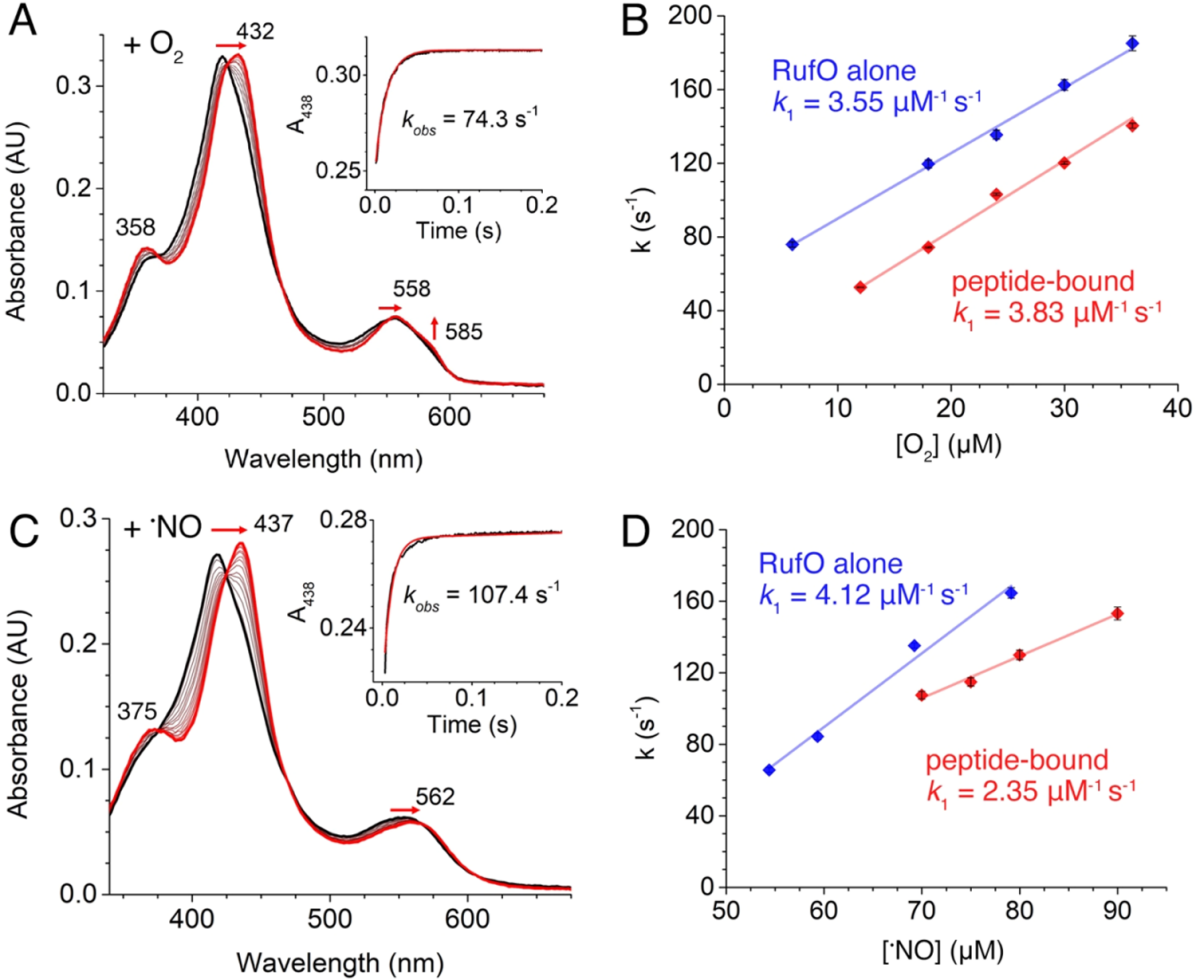
Transient kinetics of ferrous RufO binding to O_2_ and ^•^NO. Spectral changes following the rapid mixing of 3.3 μM ferrous RufO bound to MRYLH with (A) 18 μM O_2_ and (C) 70 μM ^•^NO over 0.2 s. The black-to-red spectral transitions represent the formation of (A) a ferric-superoxo intermediate and (C) a ferrous-nitrosyl complex. Insets show the time-dependent absorbance changes at 438 nm (the most pronounced shift), fitted using single exponential equations to determine rate constants (red trace). Dependence of rate constant as a function of gas concentration on (B) O_2_ and (D) ^•^NO binding to ferrous RufO alone (blue) and peptide-bound RufO (red). Second-order rate constants were determined through linear fitting.

Upon mixing with ^•^NO, a new species emerged with absorption features at 375, 437 (Soret), and 562 nm for peptide-bound ferrous RufO (Figure 5C). These spectral features are consistent with reported ferrous-nitrosyl complexes formed in CYPs.^37,48^ Similarly, mixing ferrous RufO alone with ^•^NO exhibited comparable spectral characteristics, with a Soret peak at 439 nm (Figure S9B). The rate of ^•^NO binding to ferrous RufO alone was comparable to that of O_2_, with a *k*_1_ of 4.12 μM^–1^ s^–1^, while ^•^NO binding to RufO complexed with MRYLH resulted in a lower *k*_1_ of 2.35 μM^–1^ s^–1^. Thus, O_2_ binds 1.6 times faster than ^•^NO in the presence of the peptide. Although this lower *k*_1_ is close to that observed in substrate-bound TxtE, a noticeable difference is that at low ^•^NO concentrations (<50 μM for RufO alone and <70 μM for peptide-bound RufO), the formation of the nitrosyl complexes was detectable in TxtE but not in RufO. Consequently, *k*_2_ for ^•^NO could not be estimated in RufO. This difference may arise from variations in the active site environment, as RufO has a more nonpolar distal heme pocket and lacks the I-helix kink critical for small molecule binding in TxtE and other CYPs.^22,49,50^ Therefore, ^•^NO binding to ferrous RufO is unlikely at cellular concentrations of ^•^NO, supporting the expectation that O_2_ binds first.

To further investigate whether the ferric-superoxo intermediate or the nitrosyl adduct is involved in RufO’s catalytic cycle, we performed activity assays with sequential mixing of O_2_ and ^•^NO. As shown by our LC-MS analysis (Figure S10), introduction of O_2_ followed by an equal amount of ^•^NO resulted in approximately double the product yield under single-turnover conditions compared to the reverse order. The product formation in the ^•^NO-first condition was likely due to a lower binding affinity of ^•^NO, which either left some unbound RufO before O_2_ addition or caused O_2_ to replace ^•^NO. This observation suggests a preference for O_2_ binding before ^•^NO in the catalytic sequence. Thus, the resulting ferric-superoxo intermediate is likely involved in catalysis, facilitating the subsequent activation of ^•^NO for nitration.

### Structural Insights into Substrate Recognition by RufO

Both MRYLH and Nle-RYLH peptides were successfully cocrystallized with RufO, resulting in highly similar crystal structures (Figure S11A). A root-mean-square deviation (RMSD) of 0.229 Å over 374 C_α_ atoms was determined when these two structures were superimposed. To distinguish residues from the pentapeptides and enzymes, peptide residues are labeled with a hyphen between the three-letter code and the residue number (e.g., Tyr-3), while enzyme residues are labeled without the hyphen. Aside from a minor variation in the side chains of the *N*-terminal Met-1 and Nle-1 residues, the peptide binding conformations are nearly identical (Figure S11B). Hence, a 1.51 Å resolution structure of Nle-RYLH-bound RufO was selected for presentation and discussion due to its better data quality (Table S1). This structure displays an RMSD of 0.409 Å over 316 C_α_ atoms when superimposed on the substrate-free structure. The heme binding and proximal sites show minimal structural differences, but significant conformational changes are observed in the heme distal pocket (Figure 6A). The pentapeptide-bound structure adopts a closed protein conformation, contrasting with the wide-open state of the ligand-free structure. This substantial conformational change correlates with the endothermic binding observed for the pentapeptides in the ITC experiments. The most pronounced structural differences are observed in the F-helix and G-helix, as designated in CYP structure nomenclature. This region is highly disordered in the absence of the substrate, with residues 166–181 missing and the F-helix replaced by short turns in the ligand-free form. In the complex, however, these residues become ordered, and the F-helix emerges as a cap for the distal pocket together with the extended G-helix.

**Figure 6:**
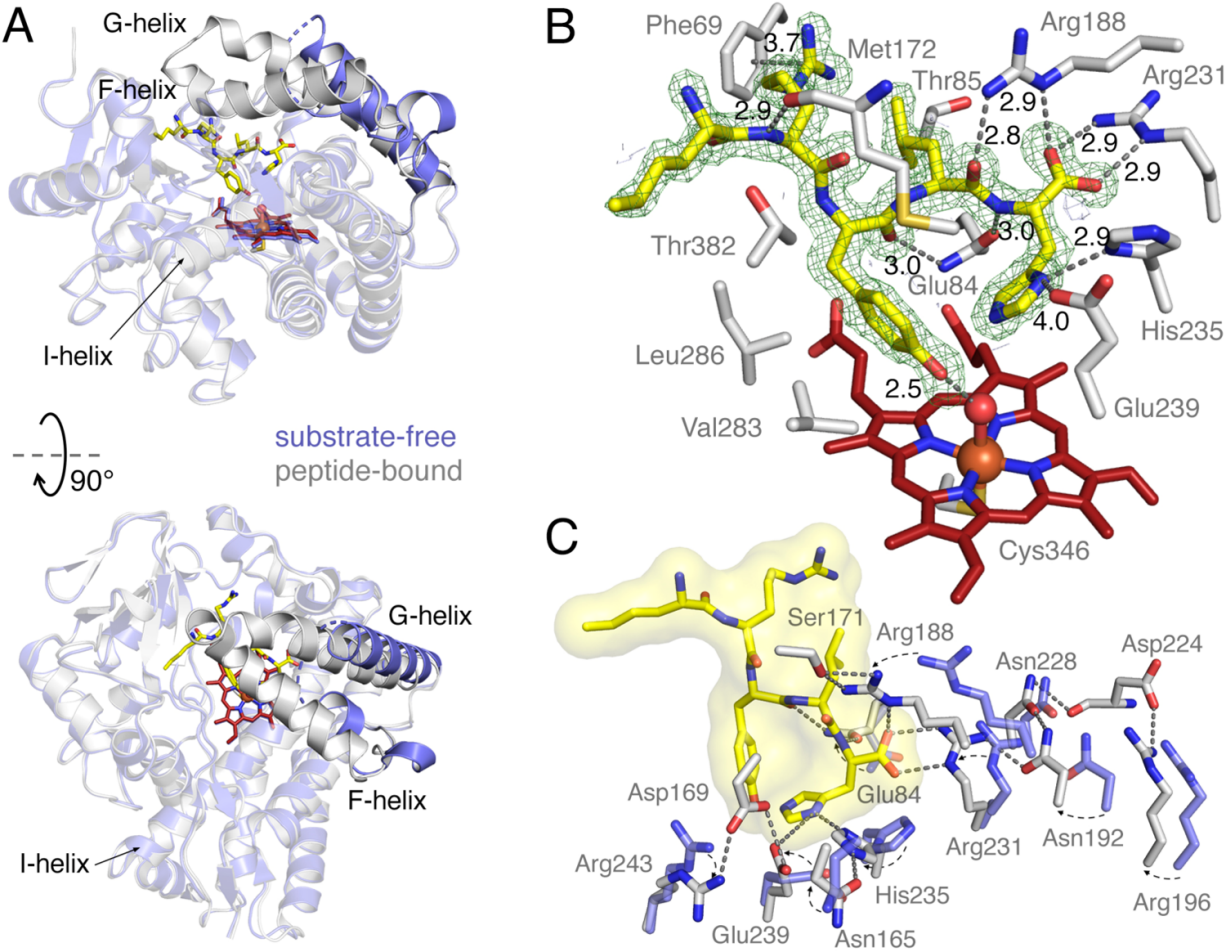
Co-crystal structure of RufO in complex with Nle-RYLH. (A) Superposition of RufO in the substrate-free form (purple, PDB code: 8SPP) and the peptide-bound form (gray, PDB code: 9DUJ). The G and F helices, which exhibit significant conformational changes, are displayed as opaque. (B) Substrate binding site showing the interactions between the active site residues and the pentapeptide. The *F*_0_–*F*_c_ omit map of the pentapeptide is represented as a green mesh and contoured at 3.0 σ. The gray dashed lines indicate distances (Å) between atoms. (C) Movements of active site residues upon peptide binding. The hydrogen-bonding network is shown by gray dashed lines. Color coding of atoms: C (substrate-free) – purple; C (peptide-bound, enzyme polypeptide) – gray; C (peptide-bound, heme) – deep red; C (peptide-bound, pentapeptide) – yellow; N – blue; O – red; S – orange; Fe – brown.

Strong and connected electron density was observed beneath the cap region and was well resolved through fitting a pentapeptide (Figure 6B). The peptide establishes most interactions via its peptide backbone and side chains of Arg-2 and His-5. Specifically, Arg-2 forms a cation-π interaction with Phe69 and a hydrogen bond with the carbonyl of Met172; amides between Tyr-3, Leu-4, and His-5, as well as the *C*-terminal carboxylate, are stabilized by H-bonding and salt bridge interactions with Glu84, Arg188, and Arg231. The imidazole N_δ_ of His-5 interacts with His235 and Glu239. Additionally, the side chain of Nle-1 occupies a hydrophobic pocket formed by surrounding nonpolar residues, and Leu-4 engages in hydrophobic interactions with Thr85. Some water molecules also bridge the pentapeptide and the protein by interacting with the guanidine of Arg-2 and the peptide backbone (Figure S12). Tyr-3, the residue targeted for nitration, is positioned above the heme center and is constrained by the hydrophobic side chains of Val283, Leu286, and Thr283. The phenol of Tyr-3 forms a short H-bond with a heme-bound water molecule modeled at half occupancy. This binding orientation aligns with our spectroscopic data showing that peptide binding does not readily displace the distal water ligand, though the close interaction induces a new low-spin heme state. Moreover, the rR spectra revealed substantial shifts in the ν­(Fe–C) and ν­(C–O) stretching modes and the δ­(Fe–C–O) bending mode upon peptide binding. These changes are likely attributable to the perturbations from the phenol of Tyr-3 on the distal CO ligand.

By comparing the substrate-free and substrate-bound RufO structures, we observed significant side chain movements in residues within the F, G, and I helices on the side interacting with the *C*-terminus of the pentapeptide (Figure 6C). These residues either interact directly with the pentapeptide or contribute to extended hydrogen-bonding networks, resulting in a constrained distal pocket. This active site rearrangement is expected to be crucial for substrate recognition and catalysis.

## Discussion

The integration of spectroscopic and structural characterization allows us to elucidate the molecular mechanism of substrate recognition and nitration activity in RufO. Our findings provide important insights into the catalytic process, enabling us to propose a mechanistic model for RufO-mediated peptide nitration (Figure S13). In its resting state, RufO adopts a six-coordinate, low-spin heme configuration with a water molecule as the distal ligand. While its heme environment is spectroscopically similar to that of typical CYPs, the flexibility of the B/C loop and the F and G helices contributes to a solvent-accessible active site in RufO that is distinct from canonical CYPs.^22^ Upon binding the MRYLH peptide substrate, the water ligand remains coordinated to the heme. A change in rhombicity upon substrate binding, as observed by EPR spectroscopy, may be attributed to a shift in the protonation state of axial solvent ligand, influenced by the nearby Tyr-3 hydroxyl group. A similar effect has been reported in CYP121, which also acts on the Tyr moiety of its substrate.^51^ Alternatively, the rhombicity change could arise from geometric distortion of the axial ligand due to steric interactions with the substrate. In support of this, the angle of the water ligand relative to the heme plane decreases from 85° in the substrate-free crystal structure to 80° in the peptide-bound crystal structure. However, this difference only results in minimal perturbations to the heme’s electronic and vibrational properties. Consequently, the reduction potential of RufO undergoes only a small increase, which likely contributes to its lack of specificity toward the tested redox partners. Nevertheless, RufO can still be reduced to the ferrous state by these electron donors. Activity assays using the prereduced enzyme and sequential additions of O_2_ and ^•^NO reveal that RufO requires only a single-electron reduction to enter the catalytic cycle. In the substrate-bound state, O_2_ associates with the reduced heme more rapidly than ^•^NO, particularly at cellular concentrations. This leads to the formation of a ferric-superoxo intermediate, as confirmed by stopped-flow spectroscopy. Subsequent ^•^NO binding likely generates a transient ferric peroxynitrite species, an intermediate that has been proposed in other heme enzymes and characterized in model systems.^37,52–54^ The peroxynitrite intermediate is expected to proceed with homolytic O–O bond cleavage, yielding a high-valent Fe­(IV)-oxo species (Compound II) and a ^•^NO_2_ radical.^52,54^ Our structural data suggest that the tyrosyl hydroxyl of the peptide substrate is positioned directly above the heme center, making it a plausible target for hydrogen atom abstraction by Compound II to generate a tyrosyl radical. This is supported by the recent report that RufO cannot nitrate the substrate analog MRFLH.^24^ Radical delocalization to the 3-position of Tyr-3 allows the radical coupling with ^•^NO_2_, ultimately yielding the nitrated pentapeptide product. While our findings strongly support the early stages of this catalytic mechanism, the formation of the ferric peroxynitrite intermediate and the generation of the tyrosyl radical require future mechanistic investigation. Notably, the production of peptide substrate is tightly regulated at the transcriptional and translational levels, and the accessibility of ^•^NO is associated with nitric oxide synthase activity. Their availability likely dictates whether catalysis proceeds or not, setting RufO apart from canonical CYPs.

The substrate-bound structures of RufO provide valuable insights into the structural basis of nitration activity and allow for a direct comparison with TxtE and other CYPs involved in RiPP biosynthesis. This comparison helps address key catalytic questions, such as how nitration activity is influenced by the active site architecture and why these enzymes exhibit divergent activity toward the same substrate. One striking difference between RufO and TxtE lies in substrate orientation and active site dynamics. In TxtE, the indole ring of Trp binds with the C4 nitration site facing away from the heme center (Figure S14), which implies that productive nitration would require substrate repositioning. This is supported by molecular dynamics simulations, which showed that TxtE undergoes loop rearrangements upon forming the reactive peroxynitrite intermediate, facilitating substrate reorientation and controlling regioselectivity.^55^ In contrast, substrate-bound RufO adopts a more confined active site. The 3-position of Tyr-3 is positioned right above the heme for radical recombination with the ^•^NO_2_ radical. The strong protein-peptide interactions in RufO impose greater conformational restriction on the substrate and reduce the extent of structural rearrangement during catalysis.

Another interesting comparison arises from P450_Blt_. Instead of catalyzing tyrosyl nitration like RufO (Figures 1 and 3A), P450_Blt_ promotes cross-linking between Tyr-3 and His-5 to form a cyclized RiPP (Figure S15), an activity also observed in CYPs involved in the biosynthesis of various biarylitides.^25,56^ In general, the peptide-bound RufO and P450_Blt_ complexes closely resemble each other, with a superposition resulting in an RMSD of 0.961 Å over 308 C_α_ atoms (Figure 7). Both enzymes bind the pentapeptide in the distal site with similar backbone conformations. However, significant differences are observed between the side chain positions of Arg-2 and His-5, which are reflected in the structural variations within the B/C-loop and F-helix that interact with Arg-2 and His-5, respectively. Arg-2 is enclosed in a hydrophobic cavity in both enzymes (Figure 7A). However, in RufO, Ile74 (in contrast to Val74 in P450_Blt_) pushes Phe69 downward into the additional space created by Ala289 (in contrast to Val287 in P450_Blt_). This difference results in the rotation of Arg-2 and its cation−π interaction with Phe69 in RufO. Although Arg-2 is positioned opposite Tyr-3 and is distant from the heme center, this interaction potentially restricts peptide dynamics during catalysis and dictates enzyme specificity.

**Figure 7:**
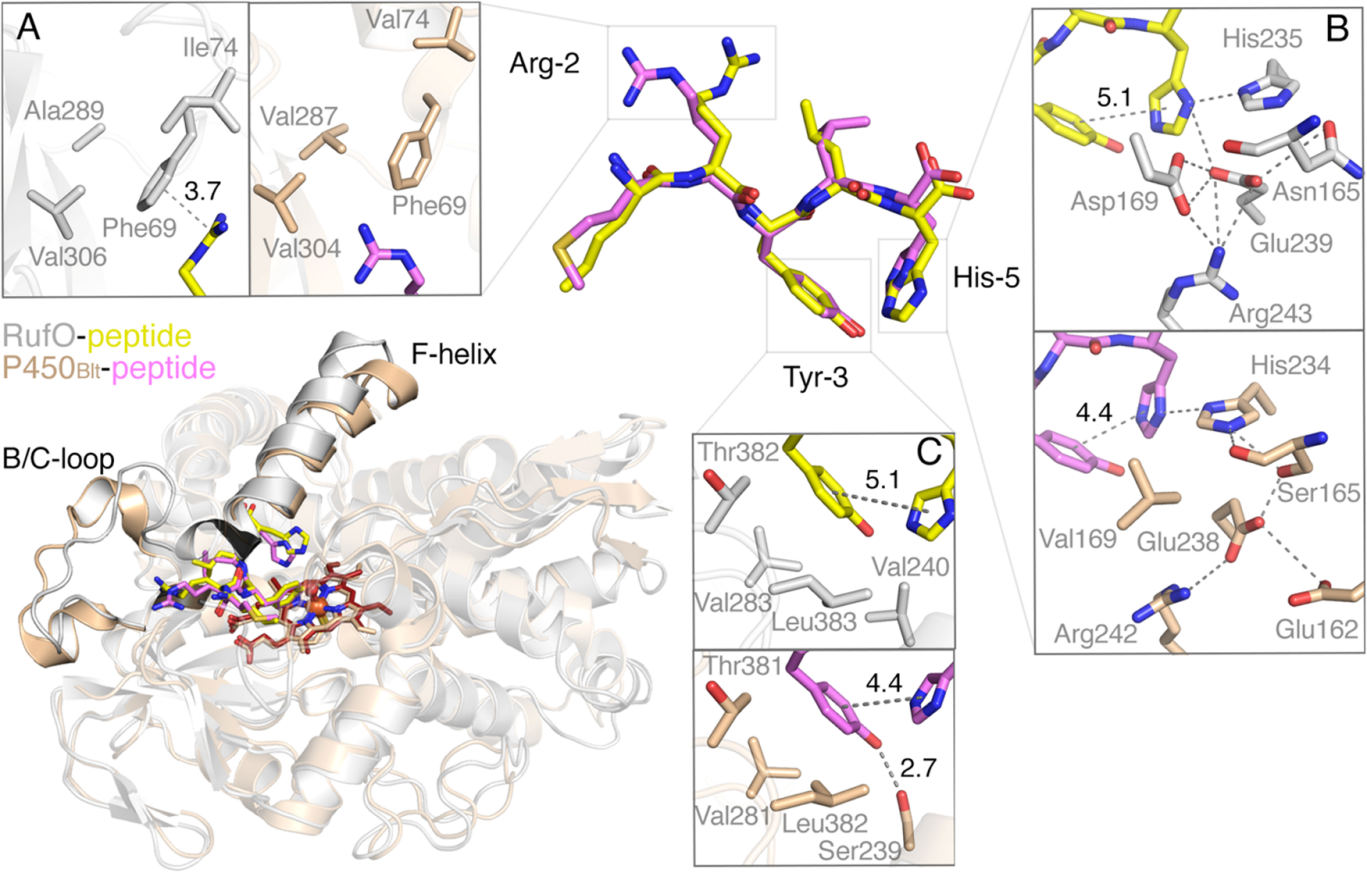
Structural comparison of pentapeptide-bound complexes of RufO and P450_**Blt**_. The superposition highlights structural differences in the B/C-loop and F-helix between RufO (shown in gray cartoon, peptide in yellow, PDB code: 9DUJ) and P450_Blt_ (shown in wheat cartoon, peptide in magenta, PDB code: 8U2M). These differences contribute to distinct side chain orientations for (A) Arg-2 and (B) His-5, while (C) Tyr-3 overlays with minimal differences in the binding cavities. Distances between Tyr-3 and His-5 in the respective structures are 5.1 and 4.4 Å.

Another important side chain movement is observed in His-5, which rotates away from Tyr-3 in RufO, increasing the distance between these two aromatic residues from 4.4 Å in P450_Blt_ to 5.1 Å in RufO (Figure 7B). This increased ring separation likely hinders peptide cross-linking in RufO. The peptide-bound complex structure of RufO predicted by molecular dynamics simulations suggests a distinct conformation of His-5, which swings away from the heme center.^24^ However, our crystal structure reveals a much more subtle movement than expected when compared to that of P450_Blt_. The ordering of the F and G helices and the connecting loop upon substrate binding to RufO shows a much more complex interaction network governs the positioning of His-5. The movement of His-5 is primarily due to the substitution of Val169 in P450_Blt_ with Asp169 in RufO. In P450_Blt_, the nonpolar Val169 disrupts the interaction between Glu238 and His-5, causing the imidazole ring to interact solely with His234 and isolating it from nearby polar residues on the F and I helices. In RufO, however, Asp169 participates in a H-bonding network centered around Glu239, which pulls out His-5 and lifts Asn165 to reposition His235. This change allows His-5 to move farther from Tyr-3. Consequently, this distinct H-bonding network leads to a conformational shift in the F-helix, rendering it more linear in RufO as opposed to the bent conformation observed in P450_Blt_. Interestingly, the key residue Tyr-3 adopts similar conformations in RufO and P450_Blt_ due to the conserved hydrophobic residues nearby (Figure 7C). However, in RufO, Tyr-3 lacks an additional H-bond due to substitution of Ser239 in P450_Blt_ with Val240, which may adjust the protonation state and redox potential of its phenol group, thereby tuning its reactivity toward heme-based intermediates. Collectively, these structural differences explain why RufO is able to catalyze nitration rather than aromatic coupling. Together with changes in active site residues involved in oxygen activation and proton relay,^24^ we now have a better picture of the structural factors dictating RufO’s unique nitration activity.

## Conclusions

The molecular basis of substrate recognition and the unique nitration activity of RufO have been comprehensively characterized using a combination of spectroscopic, kinetic, and structural techniques. MRYLH and Nle-RYLH show similar binding behaviors and nitration activities. Peptide binding to RufO is an endothermic process with high affinity. Unlike most CYPs, substrate binding in RufO does not induce the expected conversion of its six-coordinate low-spin heme to a five-coordinate high-spin heme state. Moreover, the subtle changes observed at the active site result in only a minimal increase in the reduction potential, which likely explains RufO’s lack of specificity for redox partners during nitration reactions. This lack of reductase selectivity in RufO contrasts with other CYPs, including the aromatic nitrating enzyme TxtE, which typically exhibit more defined preferences for specific electron donors. These distinctive features suggest that the early steps in RufO catalysis are already different from those in classic CYPs, and this may extend to analogous CYPs involved in RiPP biosynthesis. Substrate binding induces significant conformational changes in the distal heme pocket, where the F and G helices become ordered, facilitating substrate recognition and likely aiding in catalysis. The peptide substrate is primarily stabilized through protein interactions with the peptide main chain, Arg-2, and His-5, and residues in the F, G, and I helices undergo substantial movements to establish an extended hydrogen-bonding network with His-5. The Tyr-3 moiety to be nitrated is positioned above the heme center, forming a strong hydrogen bond with a heme-bound water molecule. This close contact results in unprecedented large shifts in vibrational frequencies originating from the CO ligand in rR spectroscopy. By comparing the peptide–protein interactions in RufO with those of a paralogous CYP catalyzing intramolecular cross-linking of the same substrate, we have gained important insights into the structural features that govern RufO’s nitration activity. Specifically, the extended hydrogen-bonding network involving His-5 found in RufO is one of the key determinants in circumventing a cross-linking reaction. Furthermore, RufO shows a preference for O_2_ binding prior to ^•^NO, resulting in a ferric-superoxo intermediate critical for the subsequent activation of *^•^* NO for nitration. Collectively, this work not only advances our understanding of how RufO functions as a peptide-nitrating enzyme but also provides broader insights into CYP-mediated RiPP biosynthesis. These findings have potential implications for the rational design of biocatalysts, particularly for applications involving selective nitration reactions and peptide modifications.

## Materials and Methods

### Preparation of RufO, TxtE, and Substrates

The expression and purification of RufO and TxtE were carried out as previously described.^22,27^ After purification, RufO samples were desalted, concentrated, aliquoted, flash-frozen in liquid nitrogen, and stored at −80 °C for future use. The protein concentrations were calculated by heme extinction coefficients of 125 and 116 mM^–1^ cm^–1^ for RufO and TxtE, respectively, determined by a hemochromogen method.^57^ The pentapeptides, MRYLH and Nle-RYLH (≥95% purity, GenScript), were dissolved in water to make 50 mM stocks.

### Isothermal Titration Calorimetry

ITC experiments were conducted at 20 °C using a MicroCal PEAQ-ITC instrument (Malvern Panalytical). RufO was prepared in a buffer containing 50 mM Tris-HCl and 100 mM NaCl at pH 7.5. The reference cell was filled with water. Each experiment involved 190 μM RufO in the sample cell, titrated with either 2.5 mM MRYLH or 2.8 mM Nle-RYLH in the syringe. Control experiments replaced the protein in the cell with buffer. The titration protocol included an initial 0.4 μL injection (excluded from analysis), followed by 19 injections of 2 μL at 2.5 min intervals. The cell was continuously stirred at 750 rpm. Data analysis was conducted using MicroCal PEAQ-ITC Analysis Software, with heats of dilution determined from the plateau region of the thermograms and subtracted from each integrated peak. All titrations are baseline corrected. The final integrated enthalpic changes were obtained by subtracting the controls from the protein-peptide titrations and fitted to a “one set of sites” binding model.

### UV–Vis Absorption Spectroscopy and EPR Measurements

RufO samples subjected to spectroscopic measurements were prepared in a buffer containing 50 mM Tris-HCl and 100 mM NaCl at pH 7.6. For the UV–vis absorption spectroscopy, MRYLH or Nle-RYLH peptide was incrementally added to 7.5 μM RufO, with a final concentration of the peptide reaching 10 μM. Spectra were recorded at room temperature using an Agilent Cary 3500 UV–vis spectrophotometer. The largest change in absorbance was detected at 409 nm (peak) and 427 nm (trough). A plot of the difference in absorbance (*A*_409_ – *A*_427_) against the peptide-to-RufO concentration ratio was generated, with linear fits applied to the slope and saturation regions to delineate a breakpoint indicative of binding stoichiometry.

EPR samples were prepared by mixing 100 μM RufO in the same buffer with 5% glycerol and either 2 mM MRYLH or Nle-RYLH, followed by flash-freezing in 4 mm quartz EPR tubes. X-band continuous-wave EPR spectra were acquired by a Bruker EMX-Plus EPR spectrometer equipped with a ColdEdge Stinger closed cycle helium flow system and an Oxford ESR900 cryostat. Each sample was measured at 9.35 GHz microwave frequency at 10 K using 1 mW microwave power. All spectra were recorded with four averaged scans using an ER4119HS resonator at a modulation frequency of 100 kHz and a modulation amplitude of 0.4 mT. The conversion rate of the low-spin species was determined by comparing the double-integrated intensities of the signals. The *g*-values were obtained by analyzing the EPR line shape.

### Activity Assays

Each assay was performed in triplicate and contained 20 μM CYP enzyme, 80 μM ferredoxin, 40 μM reductase, 1 mM substrate, 1 mM NADH/NADPH, 1 mM DEA NONOate (≥98%, Cayman Chemical), and 132 μL O_2_-saturated buffer (50 mM Tris, 100 mM NaCl at pH 8.0, bubbled for 30 min) for a final reaction volume of 200 μL. Reactions with CPR in place of the ferredoxin/reductase system contained 40 μM CPR. Reactions were incubated at 25 °C with shaking at 300 rpm for 30 min, then filtered using a 10 kDa cutoff centrifugal filter (Millipore) and analyzed by HPLC. Filtrate was analyzed on an InertSustain C18 column (5 μm particle size, 4.6 mm × 100 mm, GL Sciences Inc.) on a Thermo Fisher Scientific Ultimate-3000SD HPLC rapid separation system equipped with a photodiode array detector. The flow rate was set at 1 mL/min. For RufO assays, solvent A was water and solvent B was 90% acetonitrile (ACN) in water with 0.1% trifluoroacetic acid (TFA). A gradient of 0–70% solvent B was applied over 15 min. For collection of peptide product peaks for mass spectrometry analysis, TFA in solvent B was replaced with formic acid (FA). The method for TxtE assays used water with 0.1% FA as solvent A and ACN with 0.1% FA as solvent B. A gradient of 0–20% solvent B was applied over 10 min, followed by a 5 min hold at 20% solvent B. All presented traces were monitored at an absorbance wavelength of 280 nm. Mass spectra were collected on a Bruker Impact II quadrupole time-of-flight mass spectrometer equipped with an electrospray ionization source (Bruker Daltonics) and operated in the positive ionization mode. Samples were introduced via a loop injection with methanol as the carrier.

### Transient Kinetics of Small-Molecule Binding

Stopped-flow spectroscopic data were collected using an Applied Photophysics SX20 stopped-flow system equipped with a photodiode array detector. The system was maintained in an anaerobic chamber (O_2_ < 1 ppm) and all sample preparation was done anaerobically. Measurements were performed at 4 °C. All samples were prepared in a buffer containing 50 mM Tris-HCl and 100 mM NaCl, pH 8.0. RufO was prereduced with a minimal amount of sodium dithionite. The final mixed concentrations of RufO and MRYLH (where applicable) were 3.3 and 24 μM, respectively. Solutions of varying O_2_ concentrations were prepared by diluting air-saturated buffer with anaerobic buffer. The concentrations of O_2_ in the air-saturated buffer were measured with an Oxygraph+ oxygen electrode (Hansatech). To generate ^•^NO-containing solutions, 0.5 mM DEA NONOate (≥98%, Cayman Chemical) was incubated in anaerobic buffer for 4 h to ensure complete ^•^NO release. The ^•^NO concentrations were quantified based on a hemoglobin assay,^58^ and further dilutions were made with anaerobic buffer immediately prior to mixing. Experimental data were analyzed by the ProK software package from Applied Photophysics. The second-order rate constants were obtained from linear fitting, with the resulting equations: *y* = 3.55*x* + 54.4 for the mixing of RufO with O_2_, *y* = 3.83*x* + 6.5 for the ES complex with O_2_, *y* = 4.12*x* −158.0 for RufO with ^•^NO, and *y* = 2.35*x* – 58.9 for the ES complex with ^•^NO.

### Resonance Raman Spectroscopic Measurements

For the preparation of ferric RufO and peptide-bound complexes, 100 μM RufO was used, with or without 2 mM peptides, in a buffer containing 50 mM Tris-HCl and 100 mM NaCl at pH 7.6. rR spectra were recorded using a 405 nm excitation line from an Obis 405 nm LX SF diode laser (Coherent), with 5 mW laser power at the sample. A 405 nm ASE laser filter (Ondax) was used to eliminate the amplified spontaneous emission signal from the laser.

For ferrous-CO RufO and peptide-bound complexes, samples were prepared by reducing 100 μM RufO with 10 equiv of sodium dithionite in NMR tubes sealed with septa. The reduced samples were then purged with CO gas for 20 min, followed by the addition of 2 mM peptide under anaerobic conditions. CO was introduced for an additional 10 min before the tubes were tightly sealed. The reduction and CO addition were confirmed by the formation of the Q-band at 547 nm and the Soret band at 450 nm. Spectra were collected using a 442 nm excitation line from a He–Cd laser (Kimmon Koha), with approximately 1 mW laser power at the sample.

All rR measurements were carried out in 180° backscattering geometry, with scattered light focused by a cylindrical lens (Nikon). The beam passed through a 405 nm notch filter (Thorlabs) for ferric samples or a 442 nm notch filter (Iridian) for ferrous-CO samples, before entering an iHR550 spectrometer (Horiba Scientific). The spectrometer was configured with a 120-μm entrance slit and a 1200 grooves/mm grating (estimated spectral resolution of 10 cm^–1^) for ferric samples, or a 350-μm slit and 2400 grooves/mm grating (estimated spectral resolution of 10 cm^–1^) for ferrous-CO samples, with detection by a liquid nitrogen-cooled Symphony CCD detector (Horiba Scientific). The accuracy of absolute frequencies is about ±1 cm^–1^ based on duplicated measurements. Measurements were conducted at room temperature in 5 mm NMR tubes constantly spinning to prevent local heating and protein degradation. The acquisition times for ferric and CO-bound samples were set to 40 min and 2 h, respectively. UV–vis spectra were recorded before and after the measurements to ensure no photodegradation occurred. rR spectra were calibrated with fenchone (for all samples) and acetone-*d*_6_ (for ferrous-CO samples in high-frequency regions) and processed using GRAMS/AI software (ThermoFisher).

### Crystal Structural Determination of RufO-Peptide Complexes

RufO for crystallization was first subjected to cleavage of the His_6_-tag by tobacco etch virus (TEV) protease. RufO and TEV were mixed in a 10:1 ratio and incubated overnight at 4 °C. The untagged RufO was collected in the flowthrough from nickel-affinity purification, concentrated, and further purified through a HiLoad 16/600 Superdex 75 pg column (Cytiva) equilibrated with a buffer containing 50 mM Tris-HCl and 50 mM NaCl at pH 7.8. Upon elution, the untagged RufO was concentrated to 25 mg/mL and complexed with 1 mM substrate. The RufO Nle-RYLH cocrystal was grown in a sitting drop tray, by mixing 1.5 μL of the untagged RufO ES complex with 1.5 μL of mother liquor, followed by incubation at 5 °C. The mother liquor for this condition contained 20% (w/v) PEG3000, 100 mM Tris-HCl at pH 7.0, and 200 mM calcium acetate, and crystals appeared after 4 days. The RufO MRYLH cocrystals were prepared by a hanging drop method, incubating 2 μL of ES complex with 2 μL of mother liquor at 5 °C. The mother liquor for this condition contained 18% (w/v) PEG 3350 and 0.2 M sodium tartrate dibasic dihydrate. Crystals grew over the course of 6–8 weeks. All crystals were cryoprotected with crystallization buffer containing 20% (v/v) glycerol and flash-frozen in liquid nitrogen. The data sets were collected at 19-ID beamline at NSLS-II, Brookhaven National Laboratory, under 100 K at a wavelength of 0.97857 Å. The diffraction data were processed using HKL2000.^59^ The PDB entry of 8SPP was used as the search model for MR.^22^ The structure models were built and refined using PHENIX software packages.^60^

## Supplementary Material



## Data Availability

The crystal structures have been deposited in the RCSB Protein Data Bank with the PDB code: 9DUJ (Nle-RYLH-bound RufO) and 9EBY (MRYLH-bound RufO). Other data are contained in the article and Supporting Information.

## Notes

Additional experimental procedures covering preparation of redox proteins, determination of reduction potentials, and LC-MS analysis of sequential addition of gas molecules; X-ray crystallography data collection and refinement statistics (Table S1); spectroscopic, kinetic, and structural data (Figures S1–S13) (PDF)

## References

[1] Ongpipattanakul C., Desormeaux E. K., DiCaprio A., van der Donk W. A., Mitchell D. A., Nair S. K. (2022). Mechanism of Action of Ribosomally
Synthesized and Post-Translationally Modified Peptides. Chem. Rev..

[2] Cao L., Do T., Link A. J. (2021). Mechanisms
of action of ribosomally synthesized and
posttranslationally modified peptides (RiPPs). J. Ind. Microbiol. Biotechnol..

[3] Arnison P. G., Bibb M. J., Bierbaum G., Bowers A. A., Bugni T. S., Bulaj G., Camarero J. A., Campopiano D. J., Challis G. L., Clardy J., Cotter P. D., Craik D. J., Dawson M., Dittmann E., Donadio S., Dorrestein P. C., Entian K. D., Fischbach M. A., Garavelli J. S., Goransson U., Gruber C. W., Haft D. H., Hemscheidt T. K., Hertweck C., Hill C., Horswill A. R., Jaspars M., Kelly W. L., Klinman J. P., Kuipers O. P., Link A. J., Liu W., Marahiel M. A., Mitchell D. A., Moll G. N., Moore B. S., Muller R., Nair S. K., Nes I. F., Norris G. E., Olivera B. M., Onaka H., Patchett M. L., Piel J., Reaney M. J., Rebuffat S., Ross R. P., Sahl H. G., Schmidt E. W., Selsted M. E., Severinov K., Shen B., Sivonen K., Smith L., Stein T., Sussmuth R. D., Tagg J. R., Tang G. L., Truman A. W., Vederas J. C., Walsh C. T., Walton J. D., Wenzel S. C., Willey J. M., van der Donk W. A. (2013). Ribosomally synthesized and post-translationally
modified peptide natural products: overview and recommendations for
a universal nomenclature. Nat. Prod. Rep..

[4] Montalbán-López M., Scott T. A., Ramesh S., Rahman I. R., van Heel A. J., Viel J. H., Bandarian V., Dittmann E., Genilloud O., Goto Y., Burgos M. J. G., Hill C., Kim S., Koehnke J., Latham J. A., Link A. J., Martinez B., Nair S. K., Nicolet Y., Rebuffat S., Sahl H. G., Sareen D., Schmidt E. W., Schmitt L., Severinov K., Sussmuth R. D., Truman A. W., Wang H., Weng J. K., van Wezel G. P., Zhang Q., Zhong J., Piel J., Mitchell D. A., Kuipers O. P., van der Donk W. A. (2021). New developments
in RiPP discovery, enzymology and engineering. Nat. Prod. Rep..

[5] Richter D., Piel J. (2024). Novel types of RiPP-modifying enzymes. Curr.
Opin. Chem. Biol..

[6] Kunakom S., Otani H., Udwary D. W., Doering D. T., Mouncey N. J. (2023). Cytochromes
P450 involved in bacterial RiPP biosyntheses. J. Ind. Microbiol. Biotechnol..

[7] Denisov I. G., Makris T. M., Sligar S. G., Schlichting I. (2005). Structure
and chemistry of cytochrome P450. Chem. Rev..

[8] Meunier B., de Visser S. P., Shaik S. (2004). Mechanism of oxidation reactions
catalyzed by cytochrome p450 enzymes. Chem.
Rev..

[9] Zhong G. (2023). Cytochromes
P450 associated with the biosyntheses of ribosomally synthesized and
post-translationally modified peptides. ACS
Bio Med. Chem. Au.

[10] Kandy S. K., Pasquale M. A., Chekan J. R. (2025). Aromatic
side-chain crosslinking
in RiPP biosynthesis. Nat. Chem. Biol..

[11] Padva L., Gullick J., Coe L. J., Hansen M. H., De Voss J. J., Crusemann M., Cryle M. J. (2025). The Biarylitides: Understanding the
Structure and Biosynthesis of a Fascinating Class of Cytochrome P450
Modified RiPP Natural Products. ChemBioChem.

[12] Radi R. (2013). Protein tyrosine
nitration: biochemical mechanisms and structural basis of functional
effects. Acc. Chem. Res..

[13] de
la Lastra J. M. P., Juan C. A., Plou F. J., Pérez-Lebeña E. (2022). The nitration
of proteins, lipids and DNA by peroxynitrite derivatives-chemistry
involved and biological relevance. Stresses.

[14] Jones L. H. (2012). Chemistry
and biology of biomolecule nitration. Chem.
Biol..

[15] Groves J. T. (1999). Peroxynitrite:
reactive, invasive and enigmatic. Curr. Opin.
Chem. Biol..

[16] Bartesaghi S., Radi R. (2018). Fundamentals on the biochemistry
of peroxynitrite and protein tyrosine
nitration. Redox Biol..

[17] Barry S. M., Kers J. A., Johnson E. G., Song L., Aston P. R., Patel B., Krasnoff S. B., Crane B. R., Gibson D. M., Loria R., Challis G. L. (2012). Cytochrome
P450-catalyzed L-tryptophan
nitration in thaxtomin phytotoxin biosynthesis. Nat. Chem. Biol..

[18] Ma J., Huang H., Xie Y., Liu Z., Zhao J., Zhang C., Jia Y., Zhang Y., Zhang H., Zhang T., Ju J. (2017). Biosynthesis of ilamycins
featuring
unusual building blocks and engineered production of enhanced anti-tuberculosis
agents. Nat. Commun..

[19] Tomita H., Katsuyama Y., Minami H., Ohnishi Y. (2017). Identification
and
characterization of a bacterial cytochrome P450 monooxygenase catalyzing
the 3-nitration of tyrosine in rufomycin biosynthesis. J. Biol. Chem..

[20] Li H., Li W., Song K., Liu Y., Zhao G., Du Y. L. (2024). Nitric
oxide synthase-guided genome mining identifies a cytochrome P450 enzyme
for olefin nitration in bacterial specialized metabolism. Synth. Syst. Biotechnol..

[21] Wang X., Lin X., Jiang Y., Qin X., Ma N., Yao F., Dong S., Liu C., Feng Y., Jin L., Xian M., Cong Z. (2023). Engineering
cytochrome P450BM3 enzymes
for direct nitration of unsaturated hydrocarbons. Angew. Chem., Int. Ed..

[22] Jordan S., Li B., Traore E., Wu Y., Usai R., Liu A., Xie Z. R., Wang Y. (2023). Structural and spectroscopic characterization
of RufO indicates a new biological role in rufomycin biosynthesis. J. Biol. Chem..

[23] Dratch B. D., McWhorter K. L., Blue T. C., Jones S. K., Horwitz S. M., Davis K. M. (2023). Insights
into substrate recognition by the unusual
nitrating enzyme RufO. ACS Chem. Biol..

[24] Padva L., Zimmer L., Gullick J., Zhao Y., Sasi V. M., Schittenhelm R. B., Jackson C. J., Cryle M., Crüsemann M. (2025). Ribosomal
pentapeptide nitration for non-ribosomal peptide antibiotic precursor
biosynthesis. Chem.

[25] Zdouc M. M., Alanjary M. M., Zarazua G. S., Maffioli S. I., Crusemann M., Medema M. H., Donadio S., Sosio M. (2021). A biaryl-linked
tripeptide
from *Planomonospora* reveals a widespread class of
minimal RiPP gene clusters. Cell Chem. Biol..

[26] Isin E. M., Guengerich F. P. (2008). Substrate
binding to cytochromes P450. Anal. Bioanal.
Chem..

[27] Dodani S. C., Cahn J. K., Heinisch T., Brinkmann-Chen S., McIntosh J. A., Arnold F. H. (2014). Structural, functional, and spectroscopic
characterization of the substrate scope of the novel nitrating cytochrome
P450 TxtE. ChemBioChem.

[28] Lockart M. M., Rodriguez C. A., Atkins W. M., Bowman M. K. (2018). CW EPR parameters
reveal cytochrome P450 ligand binding modes. J. Inorg. Biochem..

[29] Mak P. J., Im S. C., Zhang H., Waskell L. A., Kincaid J. R. (2008). Resonance
Raman studies of cytochrome P450 2B4 in its interactions with substrates
and redox partners. Biochemistry.

[30] Mak P. J., Kaluka D., Manyumwa M. E., Zhang H., Deng T., Kincaid J. R. (2008). Defining resonance
Raman spectral responses to substrate
binding by cytochrome P450 from *Pseudomonas putida*. Biopolymers.

[31] Mak P. J., Gregory M. C., Sligar S. G., Kincaid J. R. (2014). Resonance Raman
spectroscopy reveals that substrate structure selectively impacts
the heme-bound diatomic ligands of CYP17. Biochemistry.

[32] Mak, P. J. Resonance Raman Spectroscopy as a Structural Probe of the Cytochrome P450 Enzymatic Cycle. In Handbook of Porphyrin Science; World Scientific Publishing Company, 2016; pp 1–120.

[33] Jing Y., Usai R., Liu Y., Kincaid J. R. (2023). Revealing substrate-induced
structural changes in active site of human CYP51 in the presence of
its physiological substrates. J. Inorg. Biochem..

[34] Yu N. T., Kerr E. A., Ward B., Chang C. K. (1983). Resonance Raman
detection of Fe-CO stretching and Fe-C-O bending vibrations in sterically
hindered carbonmonoxy ″strapped hemes″. A structural
probe of Fe-C-O distortion. Biochemistry.

[35] Uno T., Nishimura Y., Makino R., Iizuka T., Ishimura Y., Tsuboi M. (1985). The resonance
Raman frequencies of the Fe-CO stretching
and bending modes in the CO complex of cytochrome P450cam. J. Biol. Chem..

[36] Iritani Y., Ishikawa H., Mizuno M., Mizutani Y. (2023). Heme pocket structure
and its functional implications in an ancestral globin protein. Biochemistry.

[37] Louka S., Barry S. M., Heyes D. J., Mubarak M. Q. E., Ali H. S., Alkhalaf L. M., Munro A. W., Scrutton N. S., Challis G. L., de Visser S. P. (2020). Catalytic
mechanism of aromatic nitration by cytochrome
P450 TxtE: involvement of a ferric-peroxynitrite intermediate. J. Am. Chem. Soc..

[38] Martin C. P., Chen M., Martinez M. F., Ding Y., Caranto J. D. (2021). The ferric-superoxo
intermediate of the TxtE nitration pathway resists reduction, facilitating
Its reaction with nitric oxide. Biochemistry.

[39] Dangi B., Park H., Oh T. J. (2018). Effects of alternative redox partners
and oxidizing agents on CYP154C8 catalytic activity and product distribution. ChemBioChem.

[40] Li S., Du L., Bernhardt R. (2020). Redox partners:
function modulators of bacterial P450
enzymes. Trends Microbiol..

[41] Zhang W., Liu Y., Yan J. Y., Cao S. N., Bai F. L., Yang Y., Huang S. H., Yao L. S., Anzai Y., Kato F., Podust L. M., Sherman D. H., Li S. Y. (2014). New reactions and
products resulting from alternative interactions between the P450
Enzyme and redox partners. J. Am. Chem. Soc..

[42] Zuo R., Zhang Y., Jiang C., Hackett J. C., Loria R., Bruner S. D., Ding Y. (2017). Engineered
P450 biocatalysts show
improved activity and regio-promiscuity in aromatic nitration. Sci. Rep..

[43] Saroay R., Roiban G. D., Alkhalaf L. M., Challis G. L. (2021). Expanding the substrate
scope of nitrating cytochrome P450 TxtE by active site engineering
of a reductase fusion. ChemBioChem.

[44] Efimov I., Parkin G., Millett E. S., Glenday J., Chan C. K., Weedon H., Randhawa H., Basran J., Raven E. L. (2014). A simple
method for the determination of reduction potentials in heme proteins. FEBS Lett..

[45] Lewis D. F. V., Hlavica P. (2000). Interactions between redox partners
in various cytochrome
P450 systems: functional and structural aspects. Biochim. Biophys. Acta, Bioenerg..

[46] Luthra A., Denisov I. G., Sligar S. G. (2011). Spectroscopic
features of cytochrome
P450 reaction intermediates. Arch. Biochem.
Biophys..

[47] Hamdane D., Zhang H., Hollenberg P. (2008). Oxygen activation
by cytochrome P450
monooxygenase. Photosynth. Res..

[48] Quaroni L. G., Seward H. E., McLean K. J., Girvan H. M., Ost T. W., Noble M. A., Kelly S. M., Price N. C., Cheesman M. R., Smith W. E., Munro A. W. (2004). Interaction of nitric oxide with
cytochrome P450 BM3. Biochemistry.

[49] Yu F., Li M., Xu C., Wang Z., Zhou H., Yang M., Chen Y., Tang L., He J. (2013). Structural insights
into the mechanism for recognizing substrate of the cytochrome P450
enzyme TxtE. PLoS One.

[50] Schlichting I., Berendzen J., Chu K., Stock A. M., Maves S. A., Benson D. E., Sweet B. M., Ringe D., Petsko G. A., Sligar S. G. (2000). The catalytic pathway
of cytochrome P450cam at atomic
resolution. Science.

[51] Fielding A. J., Dornevil K., Ma L., Davis I., Liu A. (2017). Probing ligand
exchange in the P450 enzyme CYP121 from *Mycobacterium tuberculosis*: dynamic equilibrium of the distal heme ligand as a function of
pH and temperature. J. Am. Chem. Soc..

[52] Mondal P., Udukalage D., Mohamed A. A., Wong H. P. H., de
Visser S. P., Wijeratne G. B. (2024). A cytochrome P450 TxtE model system
with mechanistic and theoretical evidence for a heme peroxynitrite
active species. Angew. Chem., Int. Ed..

[53] Su J., Groves J. T. (2009). Direct detection
of the oxygen rebound intermediates,
ferryl Mb and NO2, in the reaction of metmyoglobin with peroxynitrite. J. Am. Chem. Soc..

[54] Sharma S. K., Schaefer A. W., Lim H., Matsumura H., Moenne-Loccoz P., Hedman B., Hodgson K. O., Solomon E. I., Karlin K. D. (2017). A six-coordinate peroxynitrite low-spin
iron­(III) porphyrinate
complex-the product of the reaction of nitrogen monoxide (·NO_g_) with a ferric-superoxide species. J. Am. Chem. Soc..

[55] Dodani S. C., Kiss G., Cahn J. K., Su Y., Pande V. S., Arnold F. H. (2016). Discovery of a regioselectivity switch
in nitrating
P450s guided by molecular dynamics simulations and Markov models. Nat. Chem..

[56] Hansen M. H., Keto A., Treisman M., Sasi V. M., Coe L., Zhao Y. W., Padva L., Hess C., Leichthammer V., Machell D. L., Schittenhelm R. B., Jackson C. J., Tailhades J., Crusemann M., De Voss J. J., Krenske E. H., Cryle M. J. (2024). Structural
insights into a side chain cross-linking biarylitide P450 from RiPP
biosynthesis. ACS Catal..

[57] Barr I., Guo F. (2015). Pyridine hemochromagen
assay for determining the concentration of
heme in purified protein solutions. Bio-Protocol.

[58] Murphy, M. E. ; Noack, E. Nitric Oxide Assay Using Hemoglobin Method. In Methods in Enzymology; Elsevier, 1994; Vol. 233, pp 240–250. 8015461 10.1016/s0076-6879(94)33027-1

[59] Otwinowski, Z. ; Minor, W. Processing of X-ray Diffraction Data Collected in Oscillation Mode. In Methods in Enzymology; Elsevier, 1997; Vol. 276, pp 307–326. 27754618 10.1016/S0076-6879(97)76066-X

[60] Adams P. D., Grosse-Kunstleve R. W., Hung L. W., Ioerger T. R., McCoy A. J., Moriarty N. W., Read R. J., Sacchettini J. C., Sauter N. K., Terwilliger T. C. (2002). PHENIX: building new software for
automated crystallographic structure determination. Acta Crystallogr., Sect. D:Biol. Crystallogr..

