# Supplementary material for: Molecular Basis for Peptide Nitration by a Novel Cytochrome P450 Enzyme in RiPP Biosynthesis
Source: ACS Catal. 2025 Jun 3;15(12):10391–404. doi: 10.1021/acscatal.5c01932 (PMC12186263; doi:10.1021/acscatal.5c01932)
Supplement: Supplementary file 1 [file cs5c01932_si_001.pdf]

Molecular Basis for Peptide Nitration by a Novel Cytochrome P450 Enzyme in RiPP Biosynthesis

Katie Nolan[1,†], Remigio Usai[1,†], Bingnan Li[1], Stephanie Jordan[1], Yifan Wang[1,*]

[1]Department of Chemistry, University of Georgia, Athens, GA 30602

[†]Equal contribution

[*]Corresponding author; email: wang.yifan@uga.edu; ORCID ID: 0000-0003-0378-2469.

Table of Contents:

Additional Experimental Procedures....2
 Expression and purification of redox proteins....2
 Determination of reduction potentials....2
 LC-MS analysis of sequential addition of O_2 and $^•NO$....2
Table S1. X-ray crystallography data collection and refinement statistics....4
Figure S1. Spectroscopic and ITC analysis of substrate binding in TxtE....5
Figure S2. UV-vis spectra of RufO with the peptide at saturating concentrations....6
Figure S3. Titration analysis of peptide Nle-RYLH binding to RufO....7
Figure S4. High-resolution MS analysis of the nitrated product from the MRYLH reaction....8
Figure S5. Activity assays using Nle-RYLH and product analysis....9
Figure S6. HPLC analysis of RufO activity assays....10
Figure S7. Redox potential measurements of RufO....11
Figure S8. Absorption spectral changes of RufO incubated with redox proteins....12
Figure S9. Transient kinetics of ferrous RufO alone binding to O_2 and $^•NO$....13
Figure S10. LC-MS analysis of RufO reactions with sequential additions of O_2 and $^•NO$....14
Figure S11. Structural comparison of RufO in complex with MRYLH and Nle-RYLH....15
Figure S12. Water bridge interactions between RufO and the pentapeptide....16
Figure S13. Proposed mechanism of peptide nitration by RufO....17
Figure S14. Active site view of TxtE shows a distinct substrate binding pose....18
Figure S15. Scheme of $P450_{Blt}$-catalyzed crosslinking reaction....19
References....20

Additional Experimental Procedures

Expression and purification of redox proteins

The PdX (#85083) and PdR (#85084) plasmids were obtained from Addgene. The genes for CPR, SpFdX, and SpFdR were cloned into pET28a expression vectors (Twist Bioscience) using methods based on previously reported protocols.[1-4] Expression and purification procedures were adapted from established protocols.[1-5]

All plasmids were transformed into *E. coli* BL21 (DE3) cells, which were cultured in Luria-Bertani medium supplemented with kanamycin (50 μg/mL) at 37 °C until OD_{600} reached 0.6. The temperature was then reduced to 20 °C, and IPTG was added to induce protein expression (0.2 mM for PdX, PdR, SpFdX, and SpFdR; 1.0 mM for CPR). For the PdX and SpFdX cultures, ferrous ammonium sulfate (20 μg/mL) was also added. The cultures were incubated for an additional 16 hours at 20 °C.

Cells were harvested by centrifugation and resuspended in Buffer A (50 mM Tris-HCl and 200 mM NaCl at pH 8.0) containing 0.1 mM phenylmethylsulfonyl fluoride (PMSF). Proteins were purified using HisTrap FF columns (Cytiva) with a gradient of Buffer B (50 mM Tris-HCl, 200 mM NaCl, and 500 mM imidazole at pH 8.0). After purification, the proteins were buffer-exchanged into 100 mM Tris-HCl, 150 mM NaCl, and 5% glycerol (pH 7.5). Purity of the protein fractions used in assays was confirmed by SDS-PAGE. Protein concentrations were determined based on the calculated A_{280} extinction coefficients.

Determination of reduction potentials

Reduction potentials were determined as previously described.[6, 7] Briefly, 9 μM RufO was incubated with 1 mM MRYLH or Nle-RYLH and 108 μM neutral red under anaerobic conditions. The samples were reduced through a reaction between xanthine and xanthine oxidase, and UV-vis spectra were recorded every 2 min for a total of 80 min at room temperature. The reduction of the heme and neutral red was monitored at 416 nm and 513 nm, respectively. To account for the interference from the dye and peptides, linear correlations of $A_{416} = 0.473A_{513} – 0.047$ and $A_{416} = 0.557A_{513} – 0.040$ were applied to correct the 416 nm absorbance in MRYLH and Nle-RYLH, respectively. The Nernst concentration term for the dye was plotted against that for the protein, and the reduction potentials of RufO were calculated using the fitted equations: $y = 0.72x + 28$ for RufO with MRYLH and $y = 0.82x – 36$ for RufO with Nle-RYLH. All reduction potentials are reported relative to the normal hydrogen electrode (NHE).

LC-MS analysis of sequential addition of O_2 and $^{\bullet}NO$

100 μM RufO was reduced by excess sodium dithionite and buffer-exchanged into 50 mM ammonium acetate buffer (pH 7.5) under anaerobic conditions, followed by the addition of 150 μM MRYLH substrate. Single-turnover reactions were conducted under two conditions: (1) 150 μM O_2 was added for 30 s, followed by 150 μM $^{\bullet}NO$ for another 30 s, and (2) 150 μM $^{\bullet}NO$ was

added for 30 s, followed by 150 μM O_2 for another 30 s. Each reaction was carried out in a total volume of 150 μL. The reactions were immediately filtered and analyzed by LC-MS. LC-MS analysis was performed using a Bruker Impact II mass spectrometer (Billerica, MA) coupled to a Bruker Elute UHPLC system. Chromatographic separation was achieved using a Thermo Hypersil-Keystone BioBasic-4 column (1 × 150 mm, 300 Å, 5 μm) with a gradient of water and acetonitrile containing 0.1% formic acid. The mass spectrometer operated in positive ion mode, scanning from 100 to 950 m/z at a rate of 1 Hz. The nebulizer pressure was set to 0.4 bar, with a dry gas flow rate of 5 L/min and a dry temperature of 200 °C.

Table S1. X-ray crystallography data collection and refinement statistics

	**Nle-RYLH-bound RufO**	**MRYLH-bound RufO**
PDB code	9DUJ	9EBY
**Data Collection**		
Wavelength (Å)	0.97857	0.97857
Space group	$P2_12_12_1$	$P2_12_12_1$
Cell dimensions		
a, b, c (Å)	56.3, 77.8, 89.2	55.2, 79.3, 88.5
α, β, ɣ (˚)	90.0, 90.0, 90.0	90.0, 90.0, 90.0
Resolution (Å)	50.00−1.51 (1.54−1.51)[a]	50.00−2.03 (2.07–2.03)
Redundancy	12.3 (10.5)	5.2 (4.1)
R_{merge}[b] (%)	11.8 (63.1)	12.1(73.8)
I/σ	45.2 (5.1)	7.9(1.4)
Completeness (%)	100.0 (100.0)	93.4 (84.1)
$CC_{1/2}$, highest resolution shell	0.93	0.508
**Refinement**		
Resolution (Å)	47.58−1.51	40.28–2.03
No. of reflections	61,898	24,147
R_{work}[c]/R_{free}[d] (%)	16.38/19.08	20.31/26.21
No. atoms/*B*-factors (Å^2)[e]		
Protein	3056/15.65	2992/26.9
Heme	43/8.67	43/16.4
Peptide ligand	50/13.64	50/24.1
Solvent	545/28.52	207/30.9
Bond lengths (Å)	0.005	0.009
Bond angles (˚)	0.870	1.049
Clash score	1.90	4.40
Ramachandran analysis		
Favored (%)	97.93	97.41
Allowed (%)	2.07	2.59
Outlier (%)	0.00	0.00

[a] Numbers in parentheses refer to data in the highest-resolution shell.

[b] $R_{merge} = \Sigma|I_h - \langle I_h\rangle|/\Sigma I_h$, where I_h is the observed intensity and $\langle I_h\rangle$ is the average intensity.

[c] $R_{work} = \Sigma||F_o| - k|F_c||/\Sigma|F_o|$.

[d] R_{free} was calculated using a subset of reflections excluded from refinement, selected by Phenix.refine with a default exclusion of 10%. The actual fractions were automatically adjusted to 3.2% for 9DUJ and 8.3% for 9EBY based on the size of the datasets.

[e] These values were calculated using a modular $B_{average}$ in CCP4.

Figure S1. Spectroscopic and ITC analysis of substrate binding in TxtE
(**A**) UV-vis spectra of 2 μM TxtE in the absence (black trace) and presence (red trace) of 0.5 mM Trp; (**B**) ITC experiment of Trp titration to TxtE. The upper panel shows the changes in differential power observed with successive injections of 2 mM Trp to 50 μM TxtE after baseline correction. The lower panel displays the integrated enthalpic changes for each injection, resulting in a fit consistent with a single binding site (red line). (**C**) Resonance Raman spectra of 100 μM TxtE in the absence (top) and presence (bottom) of 1 mM Trp.

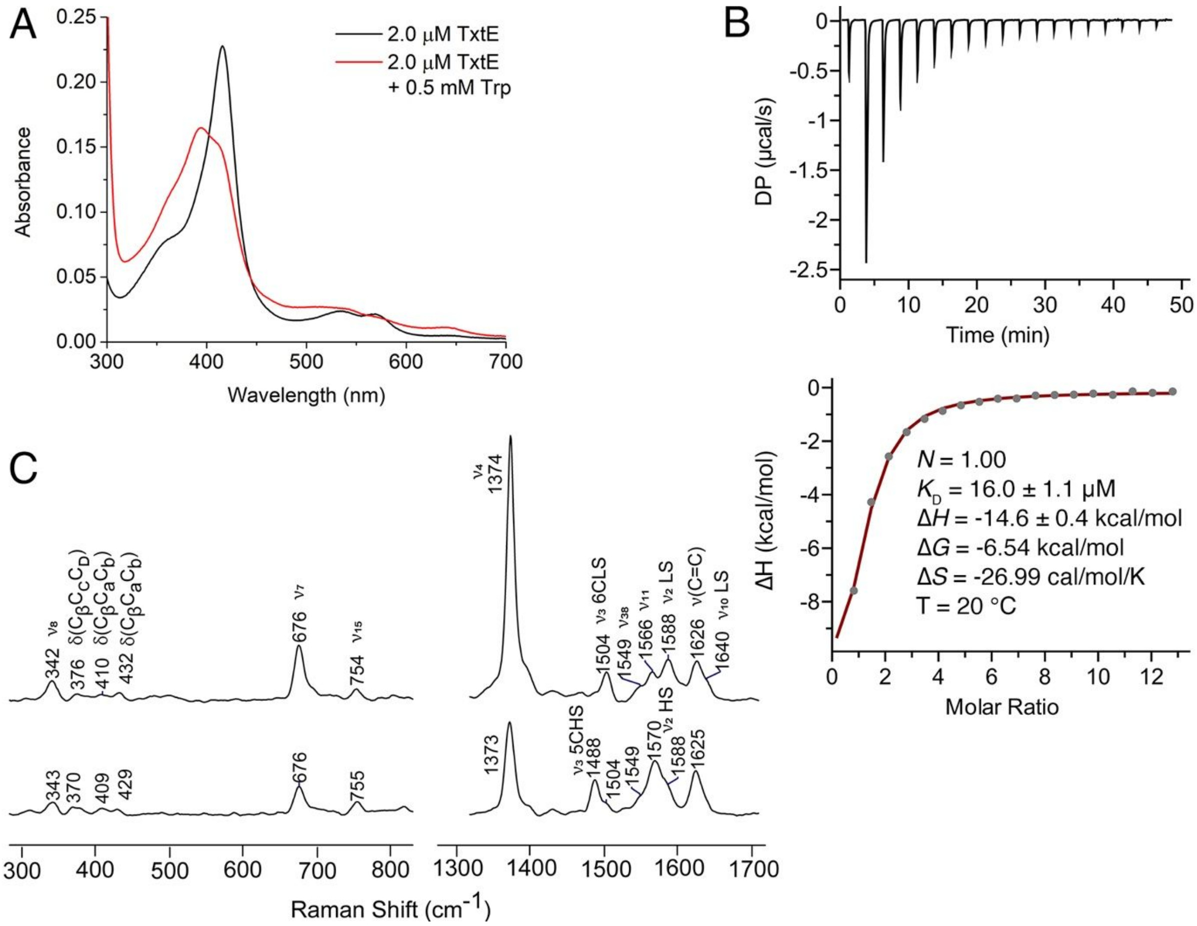

Figure S2. UV-vis spectra of RufO with the peptide at saturating concentrations
UV-vis spectra of 7.5 µM RufO alone (black), its complex with 10 µM MRYLH peptide (red), 100 µM MRYLH peptide (blue dashed line), and 200 µM MRYLH peptide (green dashed line). The unchanged spectra of the peptide-bound complexes indicate saturation beyond 10 µM concentration.

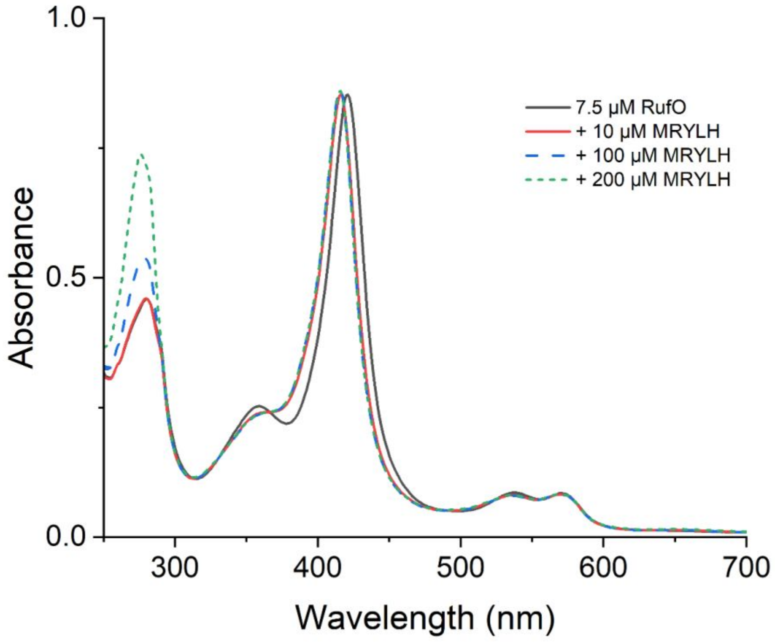

Figure S3. Titration analysis of peptide Nle-RYLH binding to RufO

(**A**) Reaction scheme of RufO-catalyzed nitration of Nle-RYLH. (**B**) UV-vis spectral changes upon titration of the peptide into 7.5 μM RufO, with the final concentration of the peptide reaching 10 μM. A transition from the resting state enzyme (blue) to the peptide-bound RufO (red) was observed. The inset represents difference spectra. (**C**) Maximum difference in absorbance (A_{409}-A_{427}) plotted against the concentration ratio of peptide to RufO. The linear fitting (red lines) indicates a binding stoichiometry of 0.68. (**D**) The upper panel shows the changes in differential power observed with successive injections of 2.8 mM peptide to 190 μM RufO (black) and the control with 2.8 mM peptide into buffer (gray). Both traces have been baseline-corrected. The lower panel displays the integrated enthalpic changes for each injection after control subtraction, resulting in a fit consistent with a single binding site (red line).

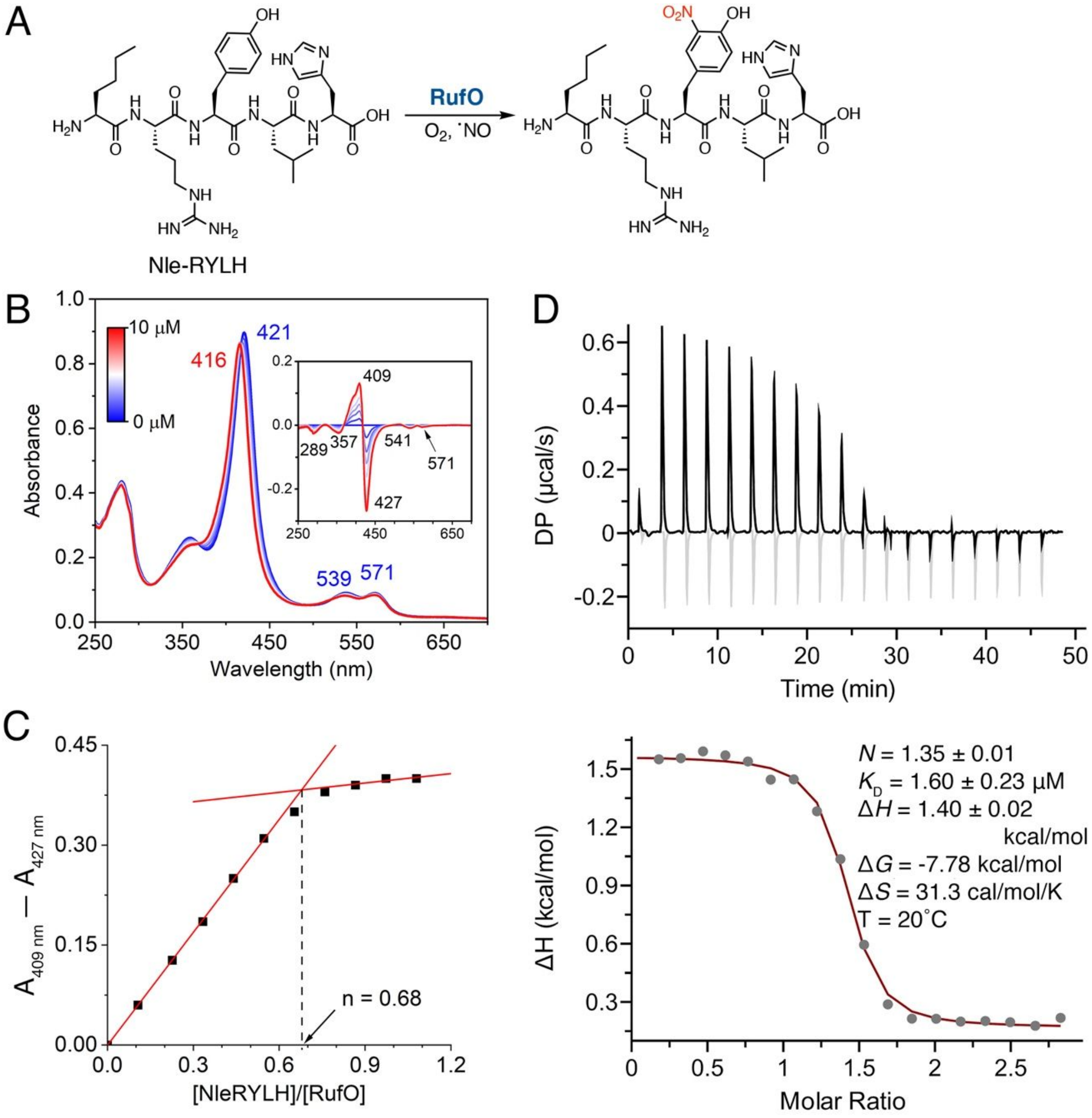

Figure S4. High-resolution MS analysis of the nitrated product from the MRYLH reaction
Comparison of experimental (top panels) and predicted (bottom panels) mass spectrometry data for the nitrated product, MR(NO_2-Y)LH, displayed as (**A**) single, (**B**) doubly, and (**C**) triply protonated ions.

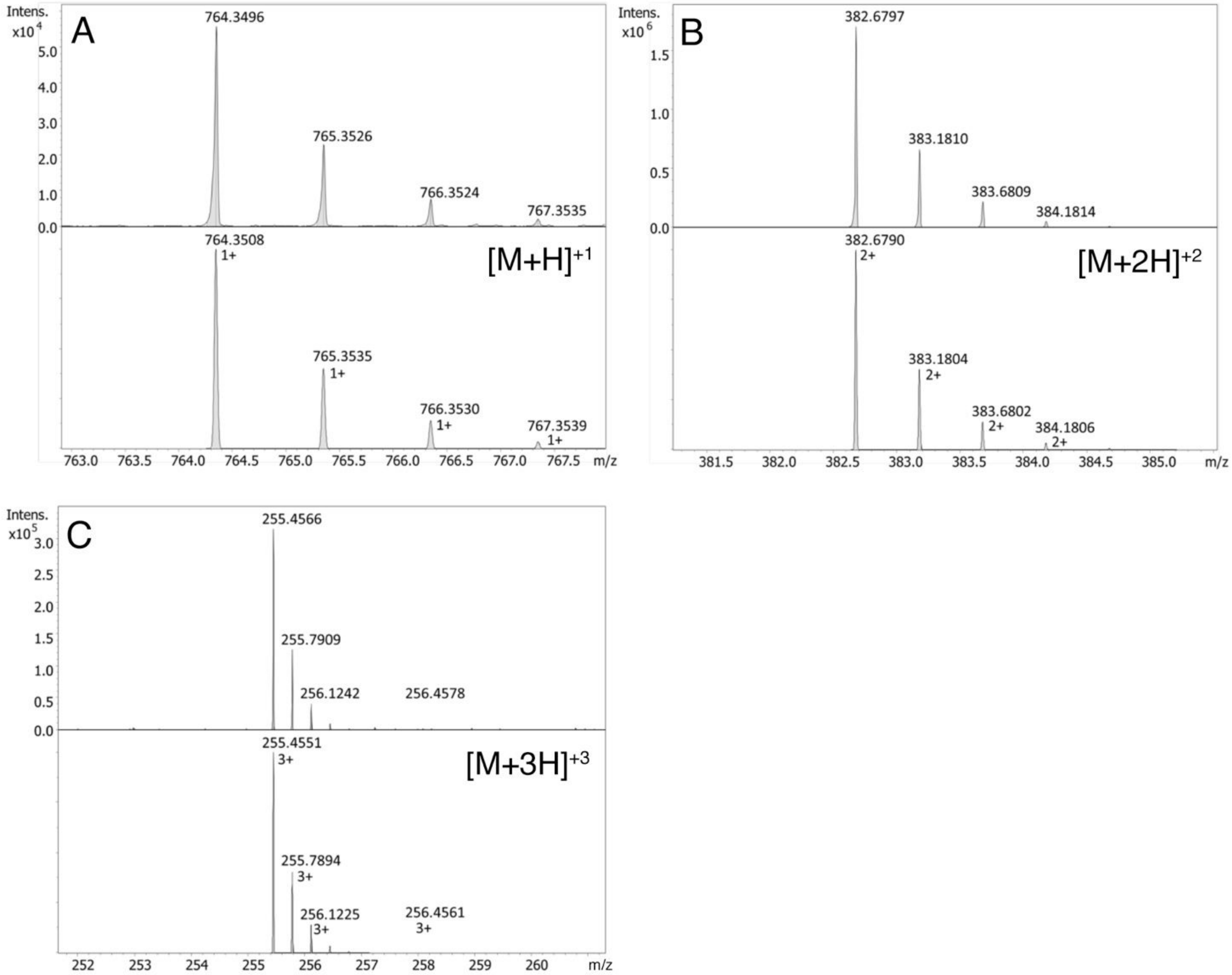

Figure S5. Activity assays using Nle-RYLH and product analysis

(**A**) HPLC profiles of (a) a reaction containing 20 μM RufO, 1 mM Nle-RYLH peptide, 1 mM NADH, 80 μM PdX, 40 μM PdR, and 1 mM DEA NONOate in the presence of O_2, (b) a control reaction without $^{\bullet}$NO addition, and (c) a control reaction without RufO addition. The pentapeptide and nitrated product eluted at 7.0 and 8.2 min, respectively. (**B**) Absorbance spectrum and (**C**) high-resolution mass spectrum of the product. (**D**-**F**) Comparison of experimental (top panels) and predicted (bottom panels) mass spectrometry data for the nitrated product displayed as single, doubly, and triply protonated ions.

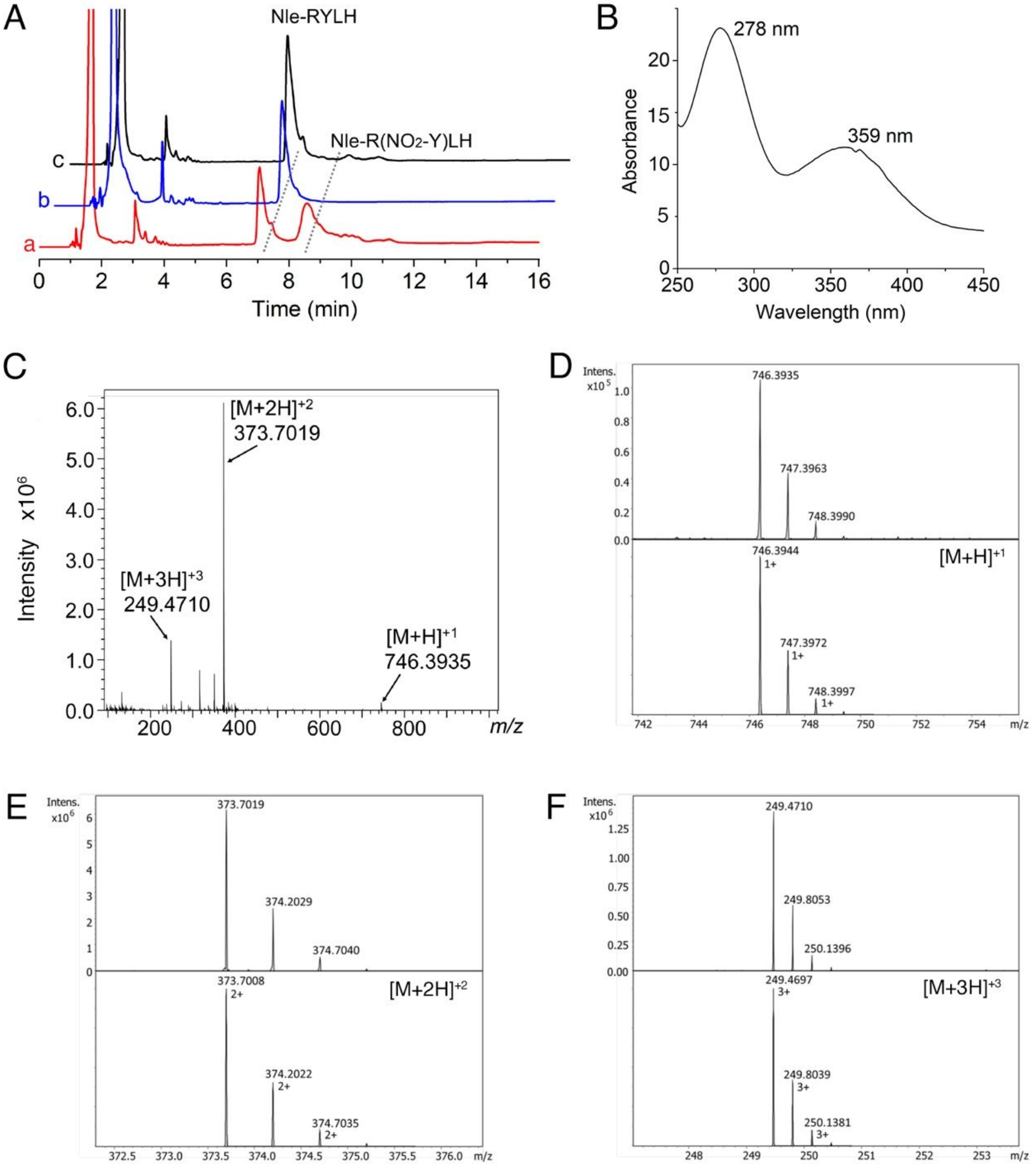

Figure S6. HPLC analysis of RufO activity assays

(**A**) HPLC profiles showing: (a) RufO reaction set up as described in the Experimental Procedures, (b) MRYLH standard, (c) reaction without •NO, (d) reaction without O_2, and (e) MRYLH incubated with O_2, •NO, and hemin. Separation was performed using an InertSustain C18 column as described in the Experimental Procedures. (**B**) HPLC profiles showing: (a) MRYLH incubated with O_2 and •NO alone and (b) MRYLH standard. Separation was performed using an Inertsil ODS-3 column (3 µm, 250 × 4.6 mm). Product formation was negligible in (a).

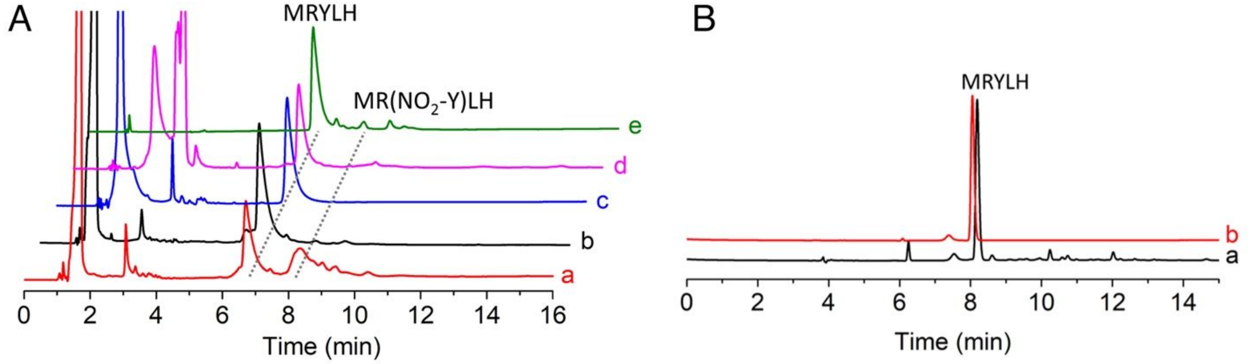

Figure S7. Redox potential measurements of RufO

Absorption spectral changes of RufO and neutral red upon reduction by xanthine oxidase in the presence of (**A**) 1 mM MRYLH and (**B**) 1 mM Nle-RYLH. Upon reduction, the heme Soret band at 416 nm blue-shifted and decreased in intensity, and the dye absorption peak at 513 nm also decreased. (**C**) Nernst plot for RufO with MRYLH (red) and Nle-RYLH (black), with data points derived from the absorption spectra. Linear fits produced equations of y = 0.72x + 28 for RufO with MRYLH and y = 0.82x + 36 for RufO with Nle-RYLH. Based on ΔE_m (y-intercept) = E_{RufO} - E_{dye} (-325 mV),[6, 7] the reduction potentials for RufO were calculated as −297 mV with MRYLH and −289 mV with Nle-RYLH.

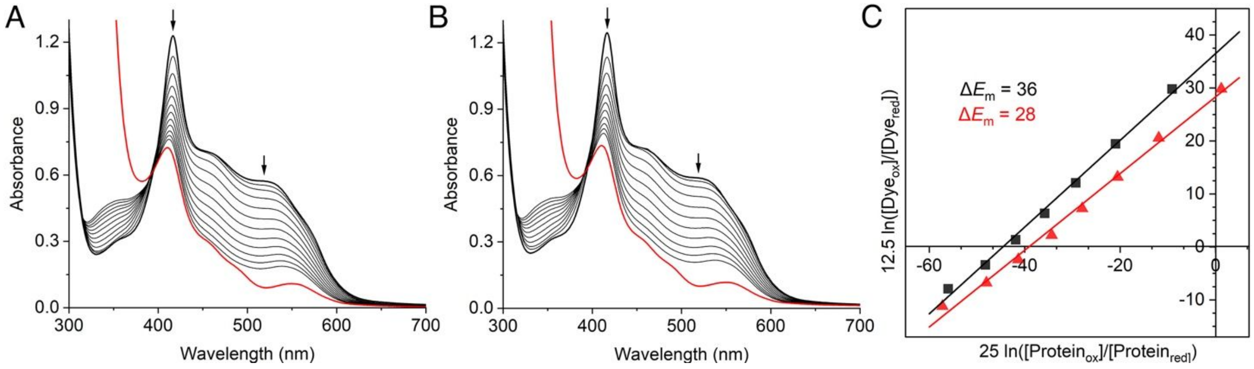

Figure S8. Absorption spectral changes of RufO incubated with redox proteins

Under anaerobic conditions, 10 μM oxidized ES complex (black) was slowly reduced to its ferrous state (red) over 30 min in the presence of 1 μM SpFdX, 1 μM SpFdR, and 400 μM NADPH. The reaction was initiated by the addition of NADPH, and the merging of the initially split Q bands (inset) suggested heme reduction.

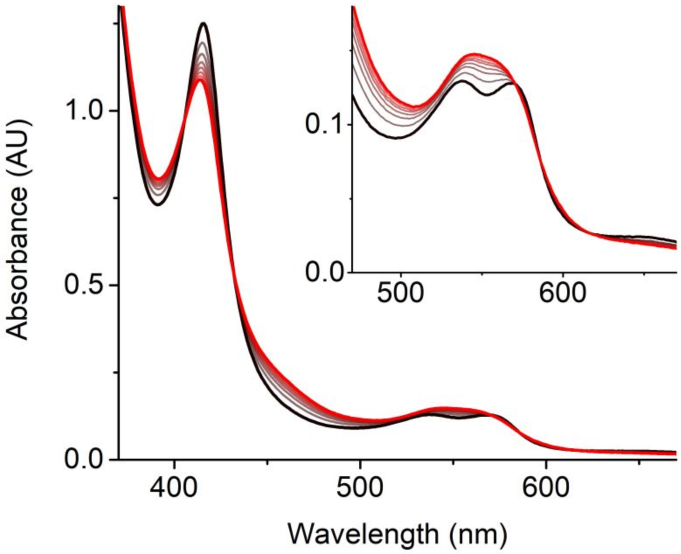

Figure S9. Transient kinetics of ferrous RufO alone binding to O_2 and $^{\bullet}NO$

Spectral changes following the rapid mixing of 3.3 μM ferrous RufO with (**A**) 18 μM O_2 and (**B**) 70 μM $^{\bullet}NO$ over 0.1 s. The black-to-red spectral transitions represent the formation of (**A**) a ferric-superoxo intermediate and (**B**) a ferrous-nitrosyl complex. Insets show the time-dependent absorbance changes at 438 nm (the most pronounced shift), fitted using first-order exponential equations to determine rate constants (red trace).

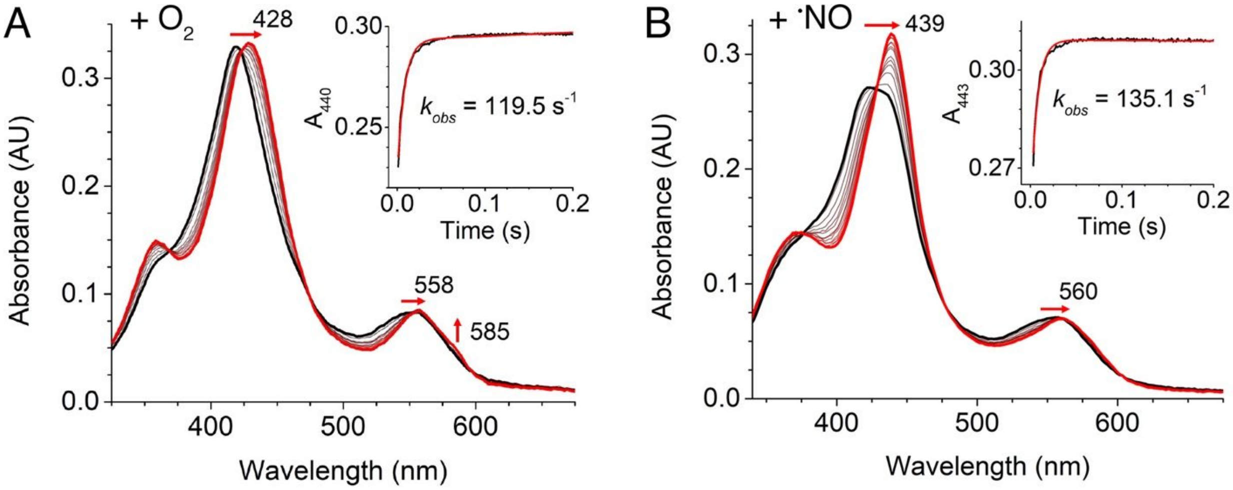

Figure S10. LC-MS analysis of RufO reactions with sequential additions of O_2 and $^{\bullet}NO$

A single-turnover reaction included 100 µM pre-reduced RufO, sequentially introduced to 150 µM O_2 or $^{\bullet}NO$ for 30 s, followed by the addition of 150 µM of the other gas for another 30 s before quenching. Extracted ion chromatograms for (**A**) O_2 then $^{\bullet}NO$ and (**B**) $^{\bullet}NO$ then O_2 reactions both show product peaks around 8.5 min, corresponding to the expected *m/z* 382.6790 for $[M+2H]^{2+}$ species. Mass spectra corresponding to the product peaks in the chromatograms for (**C**) O_2 then $^{\bullet}NO$ and (**D**) $^{\bullet}NO$ then O_2 reactions capture the $[M+2H]^{2+}$ and $[M+3H]^{3+}$ species (red labels). Collectively, the O_2-first reaction produced a higher amount of product compared to the $^{\bullet}NO$-first reaction, suggesting a preference for O_2 binding before $^{\bullet}NO$ in the catalytic sequence.

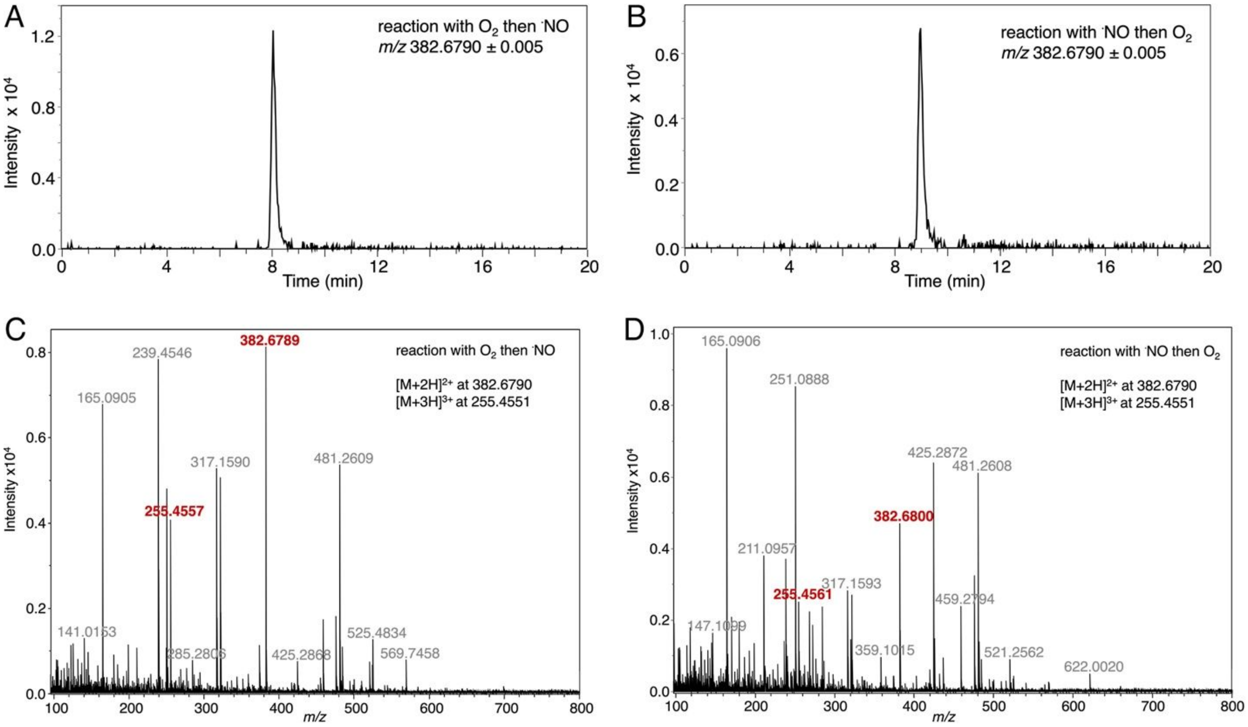

Figure S11. Structural comparison of RufO in complex with MRYLH and Nle-RYLH
(**A**) Superposition of the MRYLH-bound (green) and Nle-RYLH-bound (protein in white, peptide in yellow, and heme in red) complex structures reveals an RMSD of 0.229 Å over 374 C_{α} atoms. (**B**) Close-up view of the peptides shows a minor variation in the side chains of the terminal Met (green) and Nle (yellow) residues, while the overall binding conformations remain nearly identical.

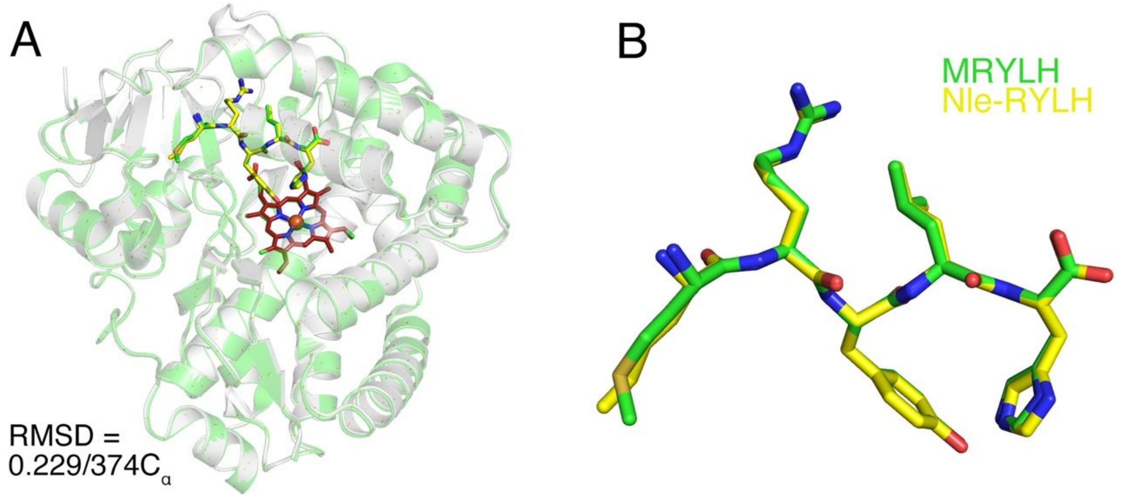

Figure S12. Water bridge interactions between RufO and the pentapeptide
The pentapeptide, Nle-RYLH, is shown as yellow sticks, protein residues as white sticks, and water molecules as red spheres. Water bridge interactions within 4.1 Å are highlighted with black lines.

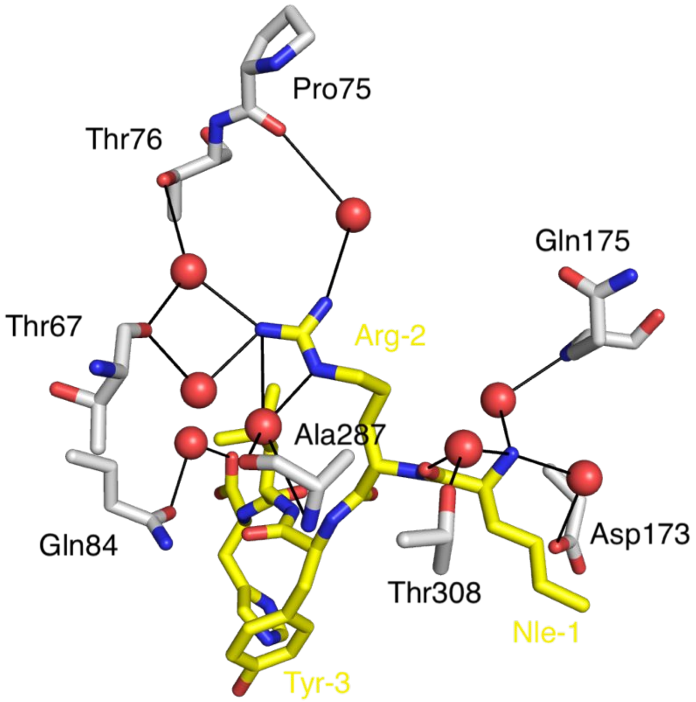

Figure S13. Proposed mechanism of peptide nitration by RufO

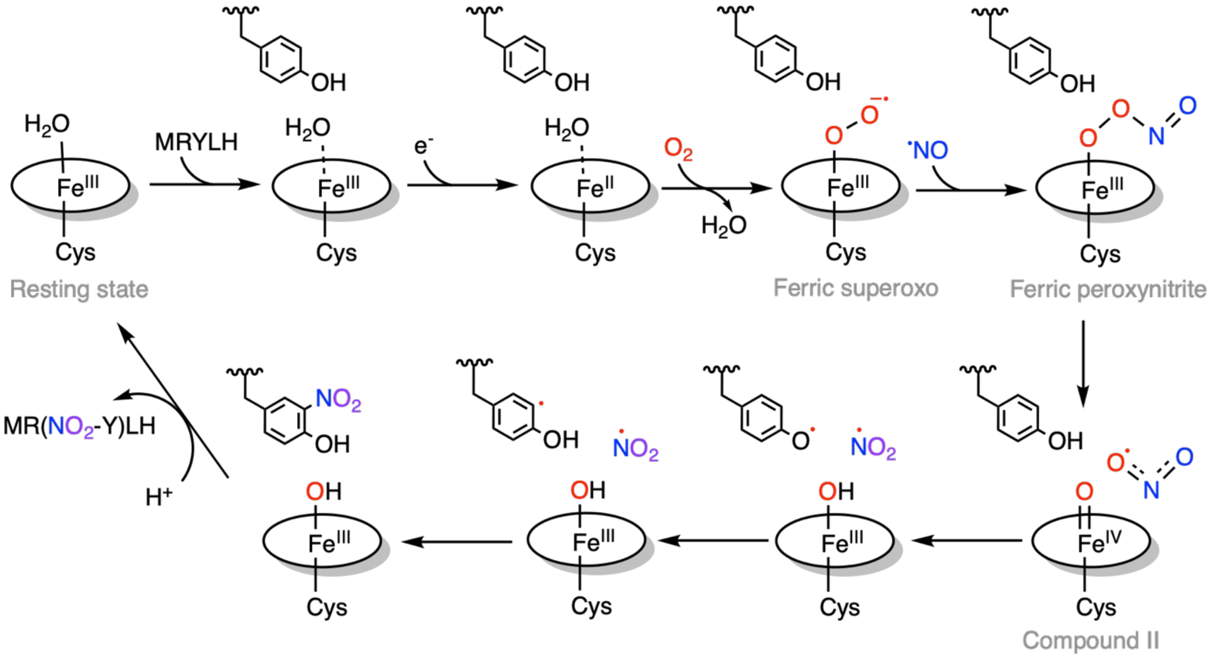

Figure S14. Active site view of TxtE shows a distinct substrate binding pose
The molecular dynamics study reveals that the disordered F/G loop plays a crucial role in determining the regioselectivity of TxtE.[8] The nitration site at C4 of the substrate Trp is indicated by the black arrow. PDB code: 4TPO.

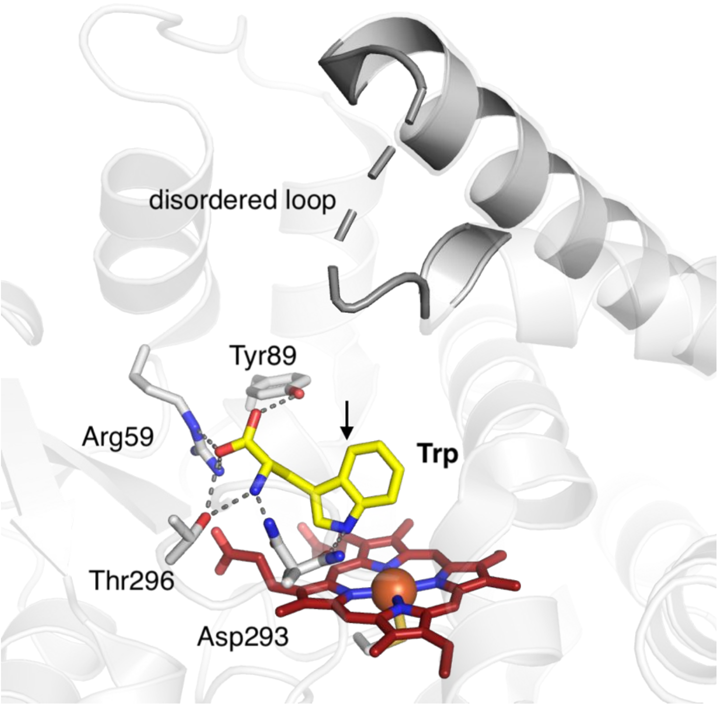

Figure S15. Scheme of P450$_{Blt}$-catalyzed crosslinking reaction

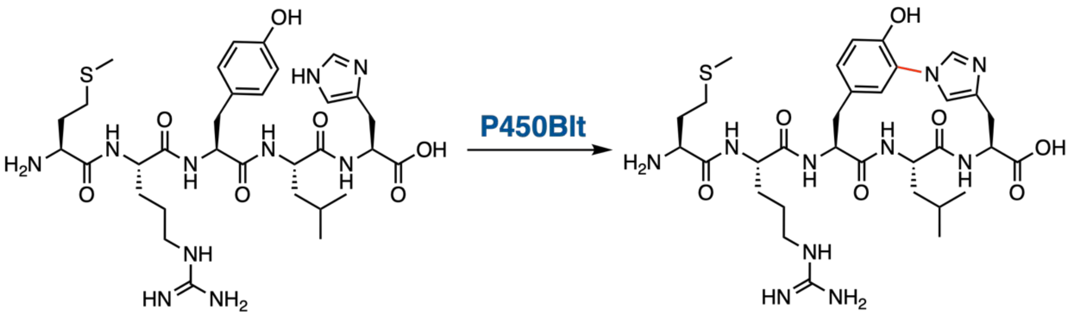

References

(1) Binda, C.; Coda, A.; Aliverti, A.; Zanetti, G.; Mattevi, A. Structure of the mutant E92K of [2Fe-2S] ferredoxin I from *Spinacia oleracea* at 1.7 Å resolution. *Acta Crystallogr. D Biol. Crystallogr.* **1998**, *54*, 1353-1358.

(2) Bruns, C. M.; Karplus, P. A. Refined crystal structure of spinach ferredoxin reductase at 1.7 Å resolution: oxidized, reduced and 2'-phospho-5'-AMP bound states. *J. Mol. Biol.* **1995**, *247* (1), 125-145.

(3) Beck von Bodman, S.; Schuler, M. A.; Jollie, D. R.; Sligar, S. G. Synthesis, bacterial expression, and mutagenesis of the gene coding for mammalian cytochrome b_5. *Proc. Natl. Acad. Sci. U. S. A.* **1986**, *83* (24), 9443-9447.

(4) Marohnic, C. C.; Panda, S. P.; Martasek, P.; Masters, B. S. Diminished FAD binding in the Y459H and V492E Antley-Bixler syndrome mutants of human cytochrome P450 reductase. *J. Biol. Chem.* **2006**, *281* (47), 35975-35982.

(5) Tan, C. Y.; Hirakawa, H.; Suzuki, R.; Haga, T.; Iwata, F.; Nagamune, T. Immobilization of a bacterial cytochrome P450 monooxygenase system on a solid support. *Angew. Chem. Int. Ed. Engl.* **2016**, *55* (48), 15002-15006.

(6) Jordan, S.; Li, B.; Traore, E.; Wu, Y.; Usai, R.; Liu, A.; Xie, Z. R.; Wang, Y. Structural and spectroscopic characterization of RufO indicates a new biological role in rufomycin biosynthesis. *J. Biol. Chem.* **2023**, *299* (8), 105049.

(7) Efimov, I.; Parkin, G.; Millett, E. S.; Glenday, J.; Chan, C. K.; Weedon, H.; Randhawa, H.; Basran, J.; Raven, E. L. A simple method for the determination of reduction potentials in heme proteins. *FEBS Lett.* **2014**, *588* (5), 701-704.

(8) Dodani, S. C.; Kiss, G.; Cahn, J. K.; Su, Y.; Pande, V. S.; Arnold, F. H. Discovery of a regioselectivity switch in nitrating P450s guided by molecular dynamics simulations and Markov models. *Nat. Chem.* **2016**, *8* (5), 419-425.
